# Supplementary material for: Study protocol for a pragmatic randomised controlled trial comparing the effectiveness and cost-effectiveness of levetiracetam and zonisamide versus standard treatments for epilepsy: a comparison of standard and new antiepileptic drugs (SANAD-II)
Source: BMJ Open. 2020 Aug 26;10(8):e040635. doi: 10.1136/bmjopen-2020-040635 (PMC7451282; doi:10.1136/bmjopen-2020-040635)
Supplement: Author's manuscript [file bmjopen-2020-040635.draft_revisions.pdf]

BMJ Open is committed to open peer review. As part of this commitment we make the peer review history of every article we publish publicly available.

When an article is published we post the peer reviewers' comments and the authors' responses online. We also post the versions of the paper that were used during peer review. These are the versions that the peer review comments apply to.

The versions of the paper that follow are the versions that were submitted during the peer review process. They are not the versions of record or the final published versions. They should not be cited or distributed as the published version of this manuscript.

BMJ Open is an open access journal and the full, final, typeset and author-corrected version of record of the manuscript is available on our site with no access controls, subscription charges or pay-per-view fees (http://bmjopen.bmj.com).

If you have any questions on BMJ Open's open peer review process please email info.bmjopen@bmj.com

BMJ Open

A study protocol for a pragmatic randomised controlled trial comparing the effectiveness and cost effectiveness of levetiracetam and zonisamide versus standard treatments for epilepsy: a comparison of Standard And New Antiepileptic Drugs (SANAD-II).

Journal:	*BMJ Open*
Manuscript ID	bmjopen-2020-040635
Article Type:	Protocol
Date Submitted by the Author:	19-May-2020

Complete List of Authors:	Balabanova, Silviya; University of Liverpool Faculty of Health and Life Sciences, Liverpool Clinical Trials Centre<br>Taylor, Claire; University of Liverpool Faculty of Health and Life Sciences, Liverpool Clinical Trials Centre<br>Sills, Graeme; University of Glasgow, School of Life Sciences<br>Burnside, Girvan; University of Liverpool Faculty of Health and Life Sciences, Biostatistics<br>Plumpton, Catrin; Bangor University, Centre for Health Economics and Medicines Evaluation<br>Smith, Phil; University Hopital of Wales, Department of Neurology<br>Appleton, Richard; Alder Hey Children's NHS Foundation Trust, Neurology<br>Leach, John; University of Glasgow, School of Medicine<br>Johnson, Michael; Imperial College London Faculty of Medicine - South Kensington Campus<br>Baker, Gus; University of Liverpool Faculty of Health and Life Sciences, Molecular and Clinical Pharmacology<br>Pirmohamed, Munir; University of Liverpool Faculty of Health and Life Sciences, Department of Pharmacology<br>Hughes, Dyfrig; Bangor University, Centre for Health Economics and Medicines Evaluation<br>Williamson, Paula; University of Liverpool, Biostatistics<br>Tudur-Smith, Catrin; University of Liverpool, Biostatistics<br>Marson, Anthony; University of Liverpool, Molecular and Clinical Pharmacology

Keywords:	Epilepsy < NEUROLOGY, Health policy < HEALTH SERVICES ADMINISTRATION & MANAGEMENT, Health economics < HEALTH SERVICES ADMINISTRATION & MANAGEMENT, Paediatric neurology < PAEDIATRICS, Clinical trials < THERAPEUTICS

SCHOLARONE™
Manuscripts

I, the Submitting Author has the right to grant and does grant on behalf of all authors of the Work (as defined in the below author licence), an exclusive licence and/or a non-exclusive licence for contributions from authors who are: i) UK Crown employees; ii) where BMJ has agreed a CC-BY licence shall apply, and/or iii) in accordance with the terms applicable for US Federal Government officers or employees acting as part of their official duties; on a worldwide, perpetual, irrevocable, royalty-free basis to BMJ Publishing Group Ltd ("BMJ") its licensees and where the relevant Journal is co-owned by BMJ to the co-owners of the Journal, to publish the Work in this journal and any other BMJ products and to exploit all rights, as set out in our licence.

The Submitting Author accepts and understands that any supply made under these terms is made by BMJ to the Submitting Author unless you are acting as an employee on behalf of your employer or a postgraduate student of an affiliated institution which is paying any applicable article publishing charge ("APC") for Open Access articles. Where the Submitting Author wishes to make the Work available on an Open Access basis (and intends to pay the relevant APC), the terms of reuse of such Open Access shall be governed by a Creative Commons licence – details of these licences and which Creative Commons licence will apply to this Work are set out in our licence referred to above.

Other than as permitted in any relevant BMJ Author's Self Archiving Policies, I confirm this Work has not been accepted for publication elsewhere, is not being considered for publication elsewhere and does not duplicate material already published. I confirm all authors consent to publication of this Work and authorise the granting of this licence.

For peer review only

TITLE PAGE

**Title**	**A study protocol for a pragmatic randomised controlled trial comparing the effectiveness and cost effectiveness of levetiracetam and zonisamide versus standard treatments for epilepsy: a comparison of Standard And New Antiepileptic Drugs (SANAD-II)**
**Authors' names**	Silviya Balabanova (0000-0001-6989-8099), Claire Taylor, Graeme Sills (0000-0002-3389-8713), Girvan Burnside, Catrin Plumpton, Phil Smith, Richard Appleton (0000-0002-0742-2113), John Paul Leach (0000-0003-2086-9937), Michael Johnson, Gus Baker, Munir Pirmohamed, Dyfrig Hughes (0000-0001-8247-7459), Paula Williamson (0000-0001-9802-6636), Catrin Tudur-Smith, Anthony Marson (0000-0002-6861-8806)
**Address for each author** Separate each author's name with a comma (don't use a separate line for each part) Please include postcodes **Authors' names and positions** Use same style of name as below title Please give one job position for each author (under the relevant address) on separate line from author's name	Clinical Trials Research Centre University of Liverpool 2nd Floor – Institute in the Park Alder Hey Children's NHS Foundation Trust Eaton Road Liverpool L12 2AP Silviya Balabanova, clinical trial co-ordinator<br>Clinical Trials Research Centre University of Liverpool 2nd Floor – Institute in the Park Alder Hey Children's NHS Foundation Trust Eaton Road Liverpool L12 2AP Claire Taylor, clinical trial co-ordinator<br>School of Life Sciences Room 346 Sir James Black Building University of Glasgow Glasgow G12 8QQ Graeme Sills, Lecturer in Pharmacology<br>Department of Biostatistics Block F Waterhouse Building University of Liverpool 1-5 Brownlow Street Liverpool L69 3GL Girvan Burnside, Senior lecturer<br>Centre for Health Economics and Medicines Evaluation Ardudwy Bangor University Holyhead Road Bangor Wales LL57 2PZ Catrin Plumpton, Research Fellow<br>The Alan Richens Epilepsy Unit University Hospital of Wales Heath Park Cardiff CF14 4XW Phil Smith, Consultant Neurologist<br>The Roald Dahl EEG Unit Alder Hey Children's Health Park Liverpool L12 2AP Richard Appleton, Consultant Paediatric Neurologist<br>School of Medicine Wolfson Medical School Building University Avenue University of Glasgow Glasgow G12 8QQ John Paul Leach, Head of Undergraduate Medicine<br>Division of Brain Science Imperial College London London SW7 2AZ Michael Johnson, Professor of Neurology and Genomic Medicine<br>Department of Molecular and Clinical Pharmacology University of Liverpool Clinical Sciences Centre Lower Lane Liverpool L9 7LJ Gus Baker, Professor of Clinical Neuropsychology<br>Department of Molecular and Clinical Pharmacology University of Liverpool Ashton Street Liverpool L69 3GE Munir Pirmohamed, Professor of Molecular and Clinical Pharmacology<br>Centre for Health Economics and Medicines Evaluation Ardudwy Bangor University Holyhead Road Bangor Wales LL57 2PZ Dyfrig Hughes, Professor of Health Economics<br>Department of Biostatistics Block F Waterhouse Building University of Liverpool 1-5 Brownlow Street Liverpool L69 3GL Paula Williamson, Professor of Biostatistics

For peer review only - http://bmjopen.bmj.com/site/about/guidelines.xhtml

	Department of Biostatistics Block F Waterhouse Building University of Liverpool 1-5 Brownlow Street Liverpool L69 3GL Catrin Tudur-Smith, Professor of Biostatistics<br>Department of Molecular and Clinical Pharmacology University of Liverpool Clinical Sciences Centre Lower Lane Liverpool L9 7LJ Anthony Guy Marson, Professor of Neurology
**Corresponding author**	Anthony Guy Marson<br>Professor of Neurology<br>Email to: marjon01@liverpool.ac.uk<br>Tel: 0151 529 5770
**Word count**	4,358

For peer review only

Keywords: epilepsy, SANAD-II, SANAD-I, antiepileptic drugs (AED), sodium valproate, levetiracetam, lamotrigine, zonisamide, treatment policy, effectiveness, cost effectiveness, health economics.

ABSTRACT

Introduction: Antiepileptic drugs (AEDs) are the mainstay of epilepsy treatment. Over the past 20 years, a number of new drugs have been approved for NHS use on the basis of information from short term trials that demonstrate efficacy. These trials do not provide information about the longer term outcomes, which inform treatment policy. This trial will assess the long term clinical and cost-effectiveness of the newer treatments levetiracetam and zonisamide.

Methods and analysis: This is a phase IV, multicentre, open-label, non-inferiority, randomised, controlled clinical trial comparing new and current standard treatments for patients with newly-diagnosed epilepsy. Arm A of the trial randomised 990 patients with focal epilepsy to standard AED lamotrigine, or the new AEDs levetiracetam or zonisamide. Arm B randomised 520 patients with generalised epilepsy to standard AED sodium valproate or new AED levetiracetam. Patients are recruited from United Kingdom (UK) NHS out-patient epilepsy, general neurology and paediatric clinics. Included patients are aged 5 years or older with two or more spontaneous seizures that require AED monotherapy, who are not previously treated with AEDs. Patients are followed-up for a minimum of 2 years. The primary outcome is time to 12-month remission from seizures. Secondary outcomes include time to treatment failure, time to treatment failure due to inadequate seizure control or due to unacceptable adverse reactions; time to first seizure; time to 24-month remission; adverse reactions and quality of life. All primary analyses will be on an intention to treat (ITT) basis. Separate analyses will be undertaken for each arm. A trial-based economic analysis will be conducted from the perspective of the NHS to assess the cost-effectiveness of each AED.

For peer review only - http://bmjopen.bmj.com/site/about/guidelines.xhtml

Ethics: All relevant trial documentation have been approved by the North West-Liverpool East Research Ethics Committee (Ref. 12/NW/0361).

Trial registration numbers: EudraCT 2012-001884-64; ISRCTN 30294119.

ARTICLE SUMMARY

Strengths and limitations of this study

- The study is adequately powered to examine the clinical effectiveness of standard and new anti-epileptic drugs (AEDs)
- The study is adequately powered to examine the cost effectiveness of standard and AEDS
- This is a long term trial which will provide evidence of the long term effects of some AEDs

- A limitation is that the study is not blinded.
- A further limitation is the reliance on patient completed questionnaires, which should be offset by access to hospital episode statistic data.

INTRODUCTION

Epilepsy is a common neurological condition and up to 3% of people will experience seizures at some time in their lives (1). Epilepsy is a complex condition with many different causes and seizures can take many different forms. It is uniquely stigmatising and has a negative impact on quality of life and employment prospects (2, 3). Antiepileptic drugs (AEDs) are the mainstay of treatment and may have to be taken for life. The ultimate goal of treatment is to maximise quality of life by eliminating seizures at drug doses that do not cause side effects. However, for many patients there is a necessary trade-off between effective seizure control and side effects, which can diminish quality of life.

Over the past 20 years, a number of new drugs have become available for the treatment of epilepsy. These new drugs have been approved for NHS use on the basis of information from short term trials. These trials do not provide information about the longer term outcomes which inform decisions made by doctors and patients, nor do they provide any useful health economic data to inform policy.

For peer review only - http://bmjopen.bmj.com/site/about/guidelines.xhtml

The SANAD-I trial began in 1999 and compared the effectiveness and cost effectiveness of standard and new treatments that were available at that time (4, 5). SANAD-I identified lamotrigine (a new drug) as an effective and cost-effective first-line treatment for patients with a focal epilepsy, and confirmed that valproate (a standard treatment) should remain a first-line drug for patients with a generalised epilepsy or seizures that clinicians find difficult to classify. Since SANAD-I, a number of newer treatments have become available, the most promising of which are levetiracetam and zonisamide. SANAD-II will include two randomised controlled trials (RCTs) run in parallel; Arm A recruiting patients with focal epilepsy and Arm B patients with generalised or unclassified epilepsy.

Rationale

Arm A

Arm A (focal epilepsy) of SANAD-II will compare lamotrigine, levetiracetam and zonisamide in patients with untreated focal onset seizures. While the focal epilepsies are further classified into a number of syndromes (6) largely according to aetiology and site of onset, it has been common practice to recruit a heterogeneous population with focal onset seizures into epilepsy trials. There are currently no reliable data that indicate whether relative treatment responses differ among the focal epilepsies, indeed prognostic modelling of data from SANAD-I suggests that treatment effects are consistent across focal epilepsy syndromes as currently classified (4, 7). In SANAD-II, we will recruit patients with focal onset seizures irrespective of syndrome, which will allow further opportunity to investigate treatment effects and factors, including syndrome, that influence those effects.

Lamotrigine was chosen as the 'standard' treatment as in SANAD-I it was found to be superior to carbamazepine for time to treatment failure and non-inferior to carbamazepine for time to 12 month remission, whilst the health economic analysis found lamotrigine a cost effective alternative to carbamazepine (4).

Levetiracetam is a commonly used antiepileptic drug with evidence for efficacy (non-inferiority to carbamazepine for 6 month seizure remission) as monotherapy in focal epilepsy from regulatory studies with too short a duration of follow-up to inform policy (8). A second industry-sponsored un-blinded trial compared levetiracetam with physicians' choice of carbamazepine or valproate, but again follow-up was too short to inform treatment policy (9).

Zonisamide is a drug that has been available for many years in Japan and other countries in South East Asia where it is commonly used both as initial monotherapy and as an add-on treatment, and is licensed for use in the EU and USA. Evidence for efficacy is from industry sponsored regulatory

For peer review only - http://bmjopen.bmj.com/site/about/guidelines.xhtml

studies demonstrating non-inferiority when compared with carbamazepine for 6 month seizure remission rates (10).

Arm B

Arm B (generalised or unclassified epilepsy) of SANAD-II will compare levetiracetam and valproate in patients with generalised onset seizures or seizures that are difficult to classify. Generalised onset seizures represent a group of syndromes, most of which are currently classified as one of the idiopathic generalised epilepsies (6), which are largely classified according to seizure type and age of onset. While differing syndromes are recognised, there is currently no reliable evidence that relative treatment responses differ among syndromes, indeed prognostic modelling of data from SANAD-I indicates that relative treatment responses are consistent across syndromes. As in SANAD-I, patients enter Arm B of the trial based upon a classification of seizures (generalised onset or difficult to classify), with patients further classified by syndrome where and when such a syndromic diagnosis can be made.

Few randomised controlled trials have been undertaken to assess the comparative effects of antiepileptic drugs in patients with generalised onset seizures, or in those with seizures that are difficult to classify, even though these individuals represent over one third of people with epilepsy. Valproate has for some time been recommended as a first line treatment for such patients (11), but without evidence from RCTs to support this recommendation. Cochrane reviews have compared valproate with other antiepileptic drugs (12, 13), but due to problems with power and epilepsy classification, none have showed an advantage for valproate. In Arm B of SANAD-I (5), valproate was significantly more effective than lamotrigine and significantly better tolerated than topiramate. Also, a double blind trial of 16 weeks therapy in childhood and juvenile absence epilepsy (14), valproate and ethosuximide were significantly superior to lamotrigine for the outcome treatment failure. Valproate however remains a difficult drug for women of childbearing potential as it is associated with a higher rate of teratogenicity than alternatives (major malformation rate ~8%) (15). There is also evidence that valproate can affect the intellectual development of children exposed in utero with up to one third of children having a significant reduction in their IQ (16). In 2018, the EMA and MHRA launched a pregnancy prevention programme for women taking valproate, and state that women of childbearing potential should not be prescribed valproate unless other treatments are ineffective or not tolerated (17). For women with generalised epilepsy, making a treatment choice is very challenging, with options including valproate, lamotrigine which is less effective but safer in pregnancy, or levetiracetam for which the efficacy when compared to valproate is unknown and for which we have increasing evidence of relative safety in pregnancy.

For peer review only - http://bmjopen.bmj.com/site/about/guidelines.xhtml

TRIAL OUTCOMES

Primary Outcome

The primary outcome of the trial is time to 12-month remission from seizures.

Secondary outcomes

The secondary outcomes are:

a. Time to treatment failure
b. Time to treatment failure due to inadequate seizure control. This event will have occurred when the clinician and/or patient decide that treatment replacement or withdrawal, or the addition of a second antiepileptic drug is required due to the occurrence of a seizure on the maximum recommended dose of randomised drug, or the maximum tolerated dose of the drug.
c. Time to treatment failure due to unacceptable adverse events. This event will have occurred when the patient experiences adverse events attributed to the drug necessitating its withdrawal.
d. Time to first seizure
e. Time to 24-month remission
f. Adverse reactions (ARs)
g. QOL
h. Health economic outcomes expressed as the incremental cost per Quality-Adjusted Life-Year (QALY) gained

TRIAL DESIGN

SANAD-II trial is a phase IV, multicentre, randomised, open-label controlled trial. Arm A of the trial recruits participants diagnosed with focal epilepsy and Arm B - with generalised or unclassified epilepsy. Trial participants are randomised to an antiepileptic drug (AED) within each Arm. In Arm A the control treatment is lamotrigine and the new treatments are levetiracetam and zonisamide, in treatment group ratio of 1:1:1. In Arm B the new treatment levetiracetam is compared to the control treatment valproate in treatment group ratio of 1:1. The sample size for the trial is 1,510 participants (990 with focal epilepsy and 520 with generalised or unclassified epilepsy).

ENROLMENT AND RANDOMISATION

Screening and baseline

The SANAD-II trial will take place in NHS out-patient epilepsy, general neurology and paediatric (epilepsy and general) clinics in the UK (a full list of participating sites is available from the trial

For peer review only - http://bmjopen.bmj.com/site/about/guidelines.xhtml

website). All patients meeting the eligibility criteria (Table 1) will be invited to participate in the study and provided with an age and development-appropriate patient information sheet and consent form. Patients will be allowed sufficient time to discuss trial and to decide on their participation. Once consent has been obtained the baseline data will be collected and patient will be progressed to randomisation.

**Table 1. Eligibility Criteria**	
Inclusion Criteria	a. Aged 5 years or older<br>b. Two or more spontaneous seizures that require antiepileptic drug treatment<br>c. Untreated and not previously treated with antiepileptic drugs, except emergency treatment in the past 2 weeks<br>d. Antiepileptic drug monotherapy considered the most appropriate option<br>e. Willing to provide consent (patients parent/legal representative willing to give consent where the patient is aged under 16 years of age or is lacking capacity to consent)
Exclusion Criteria	a. Provoked seizures only (e.g. alcohol or drug-induced)<br>b. Acute symptomatic seizures only (e.g. within 1 month from acute brain haemorrhage or brain injury or stroke)<br>c. Currently treated with antiepileptic drugs<br>d. Progressive neurological disease (e.g. known brain tumour)

Randomisation

The arm (A or B) to which the patient is assigned will be decided by the recruiting physician based upon their epilepsy classification. Patients will then be randomised to one of the following treatments: to lamotrigine, levetiracetam or zonisamide (in Arm A) or to levetiracetam or valproate (in Arm B). Randomisation will use a minimisation program with a built in random element utilising factors that will not be made known to individuals in charge of recruitment to minimise any potential for predicting allocation. Participants will be randomised using a secure (24-hour) web based randomisation programme controlled centrally by the Clinical Trials Research Centre (CTRC). Randomised treatment should begin within 7 days of randomisation. The research team should ensure that the duration between obtaining consent, performing baseline assessments, randomisation and the start of trial treatment does not impact on the well-being of the participant.

For peer review only - http://bmjopen.bmj.com/site/about/guidelines.xhtml

TRIAL INTERVENTIONS

SANAD-II is a pragmatic trial that uses market authorised drugs within the terms of marketing authorisation. All treatments will be taken as formulations already licensed to be used in UK and there will be no modifications made to the products or their outer packaging. All treatments will be prescribed as per routine NHS practice and dispensed by hospital and community pharmacies as they would normally be.

It is accepted that, for a variety of reasons including perceived or actual efficacy and tolerability, not all patients will take their medicines as prescribed. Patients will be asked about adherence in the QoL questionnaires, but no objective measurements of adherence are planned nor will the primary analyses be adjusted for actual or estimated adherence.

SANAD-II is an unblinded trial therefore decisions about concomitant medications/treatments will depend on the local medical plan and clinical management.

All patients will be titrated to an initial maintenance dose, with dose adjustments made at subsequent appointments according to the clinical response and adverse effects. Guidelines for titration and initial maintenance dose are provided within the protocol (Table 2), however clinicians will be able to alter this to choose the titration rate and initial maintenance they think most appropriate for individual patients.

**Table 2. Titration and Initial Maintenance Dose**		
**Arm A**		
Age > 12 years.		
**Lamotrigine**	**Levetiracetam**	**Zonisamide**
25mg once per day for 2 weeks<br>25mg twice per day for 2 weeks<br>50mg twice per day for 2 weeks<br>50mg morning and 100mg at night - *initial target maintenance dose*	250mg once per day for 2 weeks<br>250mg twice per day for 2 weeks<br>250mg morning and 500mg night for 2 weeks<br>500mg twice per day - *initial target maintenance dose*	50mg once per day 2 weeks<br>50mg twice per day for 2 weeks<br>50mg am 100mg pm for 2 weeks<br>100mg am 100mg pm - *initial target maintenance dose*
Children aged 5-12 years.		
**Lamotrigine**	**Levetiracetam**	**Zonisamide**
0.5 mg/kg/ day as a once a day dose for 2 weeks<br>0.5 mg/kg/day as a twice daily regimen for 2 weeks<br>0.5 mg/kg am and 1.0 mg/kg pm for 2 weeks	10 mg/kg/day as a twice daily regimen for 2 weeks<br>20 mg/kg/day as a twice daily regimen for 2 weeks<br>30 mg/kg/day as a twice daily regimen for 2 weeks	0.5 - 1 mg/kg/day as a once or twice daily regimen (depending on the child's weight) for 2 weeks<br>1 - 1.5 (maximum) mg/kg/day as a twice daily regimen for 2 weeks<br>2 - 2.5 (maximum) mg/kg/day as a twice daily regimen for 2 weeks<br>3 - 4 mg/kg/day as a twice daily

For peer review only
For peer review only - http://bmjopen.bmj.com/site/about/guidelines.xhtml

1.0 mg/kg am and 1.0 mg/kg pm for 2 weeks<br>1.5 mg/kg am and 1.5 mg/kg pm – *initial target maintenance dose*	40 mg/kg/day as a twice daily regimen – *initial target maintenance dose*	regimen for 2 weeks<br>5 mg/kg/day as a twice daily regimen - *initial target maintenance dose*.

**Arm B**	
Age > 12 years.	
**Valproate**<br>500mg once per day for 2 weeks<br>500mg twice per day - *Initial target maintenance dose*	**Levetiracetam**<br>250mg once per day for 2 weeks<br>250mg twice per day for 2 weeks<br>250mg morning and 500mg night for 2 weeks<br>500mg twice per day - *initial target maintenance dose*
Children aged 5-12 years.	
**Valproate**<br>10 mg/kg/day as a twice daily regimen for 2 weeks<br>15 mg/kg/day as a twice daily regimen for 2 weeks<br>25 mg/kg/day as a twice daily regimen – *initial target maintenance dose*	**Levetiracetam**<br>10 mg/kg/day as a twice daily regimen for 2 weeks<br>20 mg/kg/day as a twice daily regimen for 2 weeks<br>30 mg/kg/day as a twice daily regimen for 2 weeks<br>40 mg/kg/day as a twice daily regimen – *initial target maintenance dose*

The aim of treatment will be to control seizures with a minimum effective dose of drug. This will necessitate dosage modification (dose increased or reduced) if further seizures or adverse events occur as is usual clinical practice. Any changes in medication must be documented along with the justification for those changes. At the end of trial participation the participants may continue their treatment as per local policy.

To avoid potentially confounding issues, ideally patients should not be recruited into other epilepsy trials.

For peer review only

ASSESSMENTS AND PROCEDURES

Schedule for Follow up

Patients were recruited over a 4.5 year period and follow-up will continue for a further two years. The maximum time that a patient will receive their randomised treatment is 6.5 years. Table 3 shows the schedule of follow-up.

All participants will be followed up whether they are still taking their allocated treatment or not. Where patients default from clinic follow-up, additional information will be sought from General Practitioners (GP) who will be the main prescribers of AEDs in this trial. Patients will be followed up as per routine clinical practice and typically at 3, 6, and 12 months and annually thereafter. Patients may be seen at other times as clinically indicated.

For peer review only - http://bmjopen.bmj.com/site/about/guidelines.xhtml

Where treatment is stopped the participant will be asked to continue with the trial follow-up and to attend the follow-up visits.

Table 3 – Schedule of follow-up

		Follow Up Schedule		
**Procedures**	**Baseline (T0)[1]**	**T0+3 months**	**T0+6 months**	**T0+12 months and annually thereafter**
Signed Consent Form	X			
Assessment of Eligibility Criteria	X			
Contact details	X			
Review of Medical History including: • Seizure history • Neurological insult • Febrile seizures • Family history of epilepsy • EEG results • Imaging results (CT or MRI)	X			
Further investigation (EEG/ CT/ MRI)	(X)			
Allocation of Study Treatment	X			
Issue of Questionnaires in person or by post	X	X	X	X
Review of seizure occurrence & hospital admissions		X	X	X
Review of AED use (Study Treatment & Concomitant): • Since last follow up • Changes made to treatment plan including reasons		X	X	X
Assessment of Adverse Reactions		(X)	(X)	(X)
Resource use		X	X	X
Re-Issue of Questionnaire by post or at site to non-responders typically three weeks later	(X)	(X)	(X)	(X)
Telephone follow-up of questionnaire non-responders typically three weeks later	(X)	(X)	(X)	(X)
Special Assay or Procedure: Consent and obtain saliva or blood sample for later DNA analysis	(X)			

(X) – As indicated/appropriate.
1 At baseline, all procedures should be done before study intervention.

Efficacy of the trial treatments will be measured throughout the trial using a number of measures. Data on seizures will provide a subjective measure of efficacy. Quality of life data obtained

For peer review only - http://bmjopen.bmj.com/site/about/guidelines.xhtml

throughout the trial using age appropriate questionnaire booklets (Table 4) can be used as a subjective measure of efficacy.

Assessment of ARs will be undertaken at each study visit.

Quality of Life (QoL) and Utility assessments

For adults, QOL outcomes will be assessed using subscales of the NEWQOL battery and the Impact of Epilepsy Scale (18). For children and adolescents aged < 16 years, QOL assessment will involve both patient and parent-based measures: children aged 8-15 years will complete a generic health status measure validated for use in epilepsy, the KINDL (19); and the 'epilepsy impact' and 'attitude to epilepsy' subscales of the QOLIE-AD (20). Parents of all children will also complete proxy QOL questionnaires.

Utility scores will be elicited directly from trial participants (or indirectly viaparents / guardians). Adult and adolescent patients will be asked to complete the EQ-5D-3L questionnaire and Visual Analogue Scale (VAS). The EQ-5D-3L has been used previously in children, but it has not been formally validated (29), and EQ-5D-3L weights are validated for adults aged ≥18 years. The currently recommended approach of using parental proxy reports of quality of life for this age group will be used (30). EQ-5D-3L-Y (youth version) will additionally be administered to children aged 8-15 years. All trial participants will be asked to complete an epilepsy-specific utility measure, based on the NEWQOL-6D questionnaire (21).

Patients and/or parents/guardians will be completing questionnaires at baseline and at specific time points throughout the trial.

Table 4: Age-appropriate questionnaires booklets

Participant age	Questionnaire booklet completed by	Questionnaire booklet content
5 - 7	Participant	N/A
	Parent/Carer	Kiddy-KINDL, EQ-5D-3L & EQ-VAS, NEWQOL-6D
8 - 11 (Children)	Participant	Kid-KINDL, EQ-5D-3L-Y & EQ-VAS, QOLIE-AD
	Parent/Carer	Kid-KINDL, EQ-5D-3L & EQ-VAS, NEWQOL-6D
12 - 15 (Young people)	Participant	Kiddo-KINDL, EQ-5D-3L-Y & EQ-VAS, QOLIE-AD
	Parent/Carer	Kiddo-KINDL, EQ-5D-3L & EQ-VAS, NEWQOL-6D
≥ 16 (Adult)	Participant	Impact of Epilepsy Scale, EQ-5D-3L & EQ-VAS, NEWQOL-6D

Resources Use and Cost Data

Direct costs of health care resources used by patients in the trial will be collected in three ways:

For peer review only - http://bmjopen.bmj.com/site/about/guidelines.xhtml

i. A modified version of the Client Service Receipt Inventory (CSRI) (22) to assess patients' use of primary and community care services and personal social services (e.g. primary care services (NHS Direct, Walk-in treatment centres, and home visits etc.).

ii. Patients' use of secondary care services as Hospital Episode Statistics (HES) data. Downloaded Healthcare Resource Group (HRG) data will include information on out-patient epilepsy, general neurology and paediatric clinics visits; accident and emergency attendance, and length (and nature) of hospitalisations.

iii. Resources triggered by ARs will be captured in the follow-up CRF for each patient experiencing a serious AR requiring hospitalisation. Because of potential issues related to completeness of routine data, these will be used to compliment HES data.

Unit costs will be taken from the NHS reference costs database (23); and other appropriate sources (24, 25).

Genetic sub-study

DNA collection will be included as an option in SANAD-II in order that refusal will not preclude from trial participation. Whole blood or saliva samples will be shipped to a central laboratory at The University of Liverpool for extraction and storage. No genotyping of DNA samples will be undertaken as part of SANAD-II. Samples will instead be genotyped in future projects and data arising from that analysis included in international epilepsy genomics initiatives. Identification of genetic factors associated with response to treatment in epilepsy is important and may ultimately help to optimise efficacy, tolerability and safety of antiepileptic drugs.

SAMPLE SIZE CALCULATION

SANAD-II is powered to detect non-inferiority (NI) of the new antiepileptic drugs (levetiracetam and zonisamide) compared to standard treatments (lamotrigine or valproate) for the primary outcome time to 12-month remission. A new drug might become a standard first line treatment if it is proven to be non-inferior for efficacy but superior for tolerability when compared to a standard treatment – tolerability is examined in secondary outcomes including time to treatment failure for adverse effects. Powering the study for non-inferiority will also provide sufficient power to detect important differences between treatment policies.

The ILAE Commission on Antiepileptic Drugs defined limits of equivalence of +/- 10% for the primary outcome in antiepileptic drug monotherapy studies (26). However the Commission was not explicit as to whether this should be on the hazard ratio (HR) or absolute scale. No empirical work has yet been undertaken to underpin the choice of equivalence or non-inferiority margins in epilepsy trials.

For peer review only - http://bmjopen.bmj.com/site/about/guidelines.xhtml

The Chief Investigator has given numerous seminars and lectures in the UK and elsewhere about epilepsy trial methodology and the audience typically vote for a margin of 10% around absolute differences between AEDs for monotherapy studies when given examples of margins ranging from 20% to 5%.

Calculations have been informed by the SANAD-I study which estimated the 12 month remission-free probability (at 24 months) as 0.43 (exponential hazard rate of 0.0352) for lamotrigine (Arm A standard) and 0.31 (exponential hazard rate of 0.0488) for valproate (Arm B standard). The calculations assume a HR of 1.0, 80% power, and allowance for approximately 5% losses to follow-up throughout, as occurred in SANAD-I. For patients with focal-onset seizures (Arm A) two primary comparisons are of interest (levetiracetam vs lamotrigine and zonisamide vs lamotrigine), therefore the one-sided significance level has been divided by two (one-side alpha 0.0125). Assuming a 10% absolute difference in survival probability, the non-inferiority margin on the HR scale is ln(0.43)/ln(0.53)=1.329. After adjusting for 5% losses to follow-up, 330 patients are required in each of 3 treatment groups (990 total for Arm A). For patients with generalised-onset seizures or seizures that are difficult to classify (Arm B) there is only one comparison of interest (levetiracetam vs valproate). Assuming a 10% absolute difference in survival probability, the non-inferiority margin on the HR scale is ln(0.31)/ln(0.41)=1.314 for Arm B. Therefore, with a one-sided alpha of 0.025, 260 patients are required in each of two treatment groups allowing for 5% losses to follow-up (520 total for Arm B). The total number of patients required is 1510.

STATISTICAL ANALYSIS

All primary analyses will be on an intention to treat (ITT) basis including all randomised patients retained in their randomised treatment groups. Separate analyses will be undertaken for each randomisation arm. The interval (in days) from randomisation to occurrence of a 12-month remission will be summarised by Kaplan-Meier curves for each treatment group. Survival regression models will be explored; two different models will be used: (i) including the treatment effect only using treatment indicator variables; (ii) including the treatment effect together with covariates. The impact of centre effect on the treatment comparison will be investigated using both fixed and random effect models. A per protocol analysis will be undertaken to assess the robustness of ITT analyses. For Arm B trial, an additional analysis of the primary outcome will add a stratification variable to the model: seizure type (generalised / unclassified).

A similar analysis strategy will be employed for the other secondary time to event outcomes. For time to treatment failure, further analysis will be undertaken to assess the two main reasons for treatment failure - inadequate seizure control and unacceptable adverse effects. To allow for

For peer review only - http://bmjopen.bmj.com/site/about/guidelines.xhtml

possible dependence between the different withdrawal risks, cumulative incidence analyses will be presented (27).

The Haybittle-Peto approach will be employed for each interim analysis, with 99.9% confidence intervals calculated for interim analysis effect estimates. The final analysis will be undertaken at the end of the trial when all patients have a minimum two year follow-up data (six and a half years after the first patient is randomised) and 95% confidence intervals will be calculated.

QOL data will be analysed longitudinally to explore between treatments changes in scale scores over time, taking account of baseline QOL.

For the analysis of adverse reactions, all patients who received any amount of each study drug will be included in the safety analysis dataset in the treatment group they actually received. All adverse reactions and serious adverse reactions reported by the clinical investigators will be presented, identified by treatment group. Adverse reactions will be grouped according to a pre-specified coding system and tabulated. The number (and percentage) of patients experiencing each AR, and the number (and percentage) of occurrences of each AR will be presented. No formal statistical testing will be undertaken.

HEALTH ECONOMIC EVALUATION

For the health economic analyses, the perspective of the NHS and Personal Social Services will be adopted for costing purposes. It will be assessed whether levetiracetam or zonisamide as monotherapy in newly-treated focal epilepsy are cost-effective by estimating the incremental cost-utility and cost-effectiveness ratios relative to lamotrigine and to each other. The same approach will be used to compare levetiracetam and valproate for generalised-onset seizures. A cost consequence analysis (28) will be conducted to consider non-health benefits which are neither captured within the QALY calculation, nor in the cost-effectiveness analyses. Potential non-health benefits that will be measured include: social activity, time in work or school and patients' driving (captured in the NEWQOL battery). Additional non-health benefits (perceived stigma, control, and cognitive effects) will also be captured in the NEWQOL-6D (21).

Sensitivity analyses will be conducted to test the robustness of our findings. These analyses will be based on the observed distributions of outcome and costs to test whether, and to what extent, the incremental cost-utility and cost-effectiveness ratios are sensitive to key assumptions in the analysis. Uncertainty in parameter estimates will be addressed through the application of bootstrapping and the estimation of cost-effectiveness acceptability curves.

For peer review only - http://bmjopen.bmj.com/site/about/guidelines.xhtml

The estimated incremental cost per QALY will be compared with the threshold for cost-effectiveness operating in the UK, and the incremental cost per seizure avoided and per 12-month remission compared with results of other economic assessments of antiepileptic drugs (29, 30).

ETHICS and Dissemination

Ethics

The trial protocol complies with the SPIRIT reporting guidelines (31). All relevant trial documentation have been approved by the North West - Liverpool East Research Ethics Committee (Ref: 12/NW/0361). This trial falls within the remit of the EU Directive 2001/20/EC (32), transposed into UK law as the UK Statutory Instrument 2004 No 1031: Medicines for Human Use (Clinical Trials) Regulations 2004 as amended (33). SANAD-II has been registered with the MHRA and was granted a Clinical Trial Authorisation (CTA) for Notification prior to initiation.

Dissemination

The trial team plan to disseminate the SANADII results by presentations at national (e.g. ABN) and international (e.g. ILAE – European congress, or ILAE international Epilepsy Congress) as well as publications in leading peer reviewed journals. Authorship of any documents will follow ICMJE Recommendations 2018.

DATA COLLECTION AND TRIAL MONITORING

Data collection

Data management for SANAD-II is performed by the CTRC at University of Liverpool. Participating centres will be expected to each maintain a file of essential trial documentation (Site File), which will be provided by the coordinating centre, and keep copies of all completed Case Report Forms (CRFs). Data collection will use paper CRFs and participant completed questionnaires.

Trial Monitoring

Trial Oversight Committees have been formed in relation to the monitoring. This includes an Independent Data and Safety Monitoring Committee (IDSMC), consisting of independent epilepsy experts and statisticians; A Trial Management Group (TMG) consisting of the Chief Investigator, trial manager, trial statisticians, sponsor representatives, and several principal investigators, and a Trial

For peer review only - http://bmjopen.bmj.com/site/about/guidelines.xhtml

Steering Committee (TSC) consisting of two independent epilepsy clinicians, an independent statistician and a lay representative, in addition to select members of the TMG.

Trial monitoring is informed by a risk assessment to determine the level and type of monitoring required for specific hazards. The IDSMC will monitor participant safety including any unexpected adverse reactions. Adverse reactions will be reported to the MHRA and to the Liverpool East Research Ethics Committee via annual Data and Safety Monitoring Reports (DSURs). The committees will also monitor recruitment rates and adherence to GCP.

Patient and Public Involvement (PPI) Statement

SANAD II was designed in collaboration with Epilepsy Action and has a PPI representative in the Trial Steering Committee.

TIME FRAME AND TRIAL STATUS

The study opened to recruitment in 2013 and completed recruitment in June 2017. Participants are followed-up for a minimum of 2 years and a maximum of 6.5 years. Follow-up visits and data collection will continue until June 2019.

Author Affiliations

University of Liverpool, University of Glasgow, Bangor University, University Hospital of Wales, Alder Hey Children’s Health Park, Imperial College London

Acknowledgments

We would like to acknowledge the input of the wider SANAD-II team and to thank the research sites and participants for being involved in this study. We would like to specifically thank Epilepsy Action for their invaluable contribution throughout the trial.

Author Contributions

GS, PS, RA, JL, MJ, G Baker, MP, DH, PW, CTS and AM were involved in the trial design and the grant application preparation. SB, GS, G Burnside, CP, PS, RA, JL, MJ, G Baker, MP, DH, PW, CTS and AM were involved in the protocol development and protocol amendments throughout the trial. SB, CT and AM were responsible for the drafting of the manuscript. All authors were able to contribute to the draft and approved the final text.

For peer review only - http://bmjopen.bmj.com/site/about/guidelines.xhtml

Trial Sponsors

The SANADII trial is co-sponsored by the University of Liverpool (Sponsor Representative Alex Astor [sponsor@liverpool.ac.uk]) and The Walton Centre (Sponsor Representative Martin Wilby [RDI@thewaltoncentre.nhs.uk]).

Funding statement

This work is supported by the Health Technology Assessment Programme (HTA) of National Institute for Health Research (NIHR) grant number 09/144/09.

Competing Interests

None declared.

Provenance and peer review

The study, this protocol v8.0 dated 28/11/2018 and all superseded versions, and related trial documents have been approved by a registered Research Ethics Committee. The study was peer reviewed during the funding application process to the NIHR HTA programme.

Confidentiality and Data Sharing Statement

Identifiable details of those participating in the trial will remain confidential and accessible only to researchers at a patient's hospital site. The information collected by hospital site for the purpose of the study may be provided to researchers running other research studies. These researchers may be universities, NHS organisations or companies involved in health and care research in this country or abroad. The information will only be used by organisations and researchers to conduct research in accordance with the UK Policy Framework for Health and Social Care Research. This information will not identify any participants and will not be combined with other information in a way that could identify participants.

Upon completion of SANADII the trial results will be disseminated at scientific and/or medical conferences, through peer-reviewed publications, and will be made available on the study website.

Open Access

This is an Open Access article.

For peer review only

REFERENCES

1. Hauser WA HD. Epilepsy: frequency, causes and consequences. : New York: Demos Publications; 1990.
2. Holland P, Lane S, Whitehead M, Marson AG, Jacoby A. Labor market participation following onset of seizures and early epilepsy: Findings from a UK cohort. Epilepsia. 2009;50(5):1030-9.
3. Schachter SC. Quality of life for patients with epilepsy is determined by more than seizure control: the role of psychosocial factors. Expert review of neurotherapeutics. 2006;6(1):111-8.
4. Marson AG, Al-Kharusi AM, Alwaidh M, Appleton R, Baker GA, Chadwick DW, et al. The SANAD study of effectiveness of carbamazepine, gabapentin, lamotrigine, oxcarbazepine, or topiramate for treatment of partial epilepsy: an unblinded randomised controlled trial. Lancet. 2007;369(9566):1000-15.
5. Marson AG, Al-Kharusi AM, Alwaidh M, Appleton R, Baker GA, Chadwick DW, et al. The SANAD study of effectiveness of valproate, lamotrigine, or topiramate for generalised and unclassifiable epilepsy: an unblinded randomised controlled trial. Lancet. 2007;369(9566):1016-26.
6. Commission on Classification and Terminology of the International League Against Epilepsy. Proposal for revised classification of epilepsies and epileptic syndromes. In: Epilepsy. CoCaTotILA, editor. Epilepsia1989 Jul-Aug. p. 389-99.
7. Bonnett L TSC, Williamson PR, Marson AG. Developing a Prognostic Model for Epilepsy Data. 31st Annual Conference of the International Society for Clinical Biostatisticians, ISCB; Montpellier2010. p. 145.
8. Brodie MJ, Perucca E, Ryvlin P, Ben-Menachem E, Meencke HJ, Levetiracetam Monotherapy Study G. Comparison of levetiracetam and controlled-release carbamazepine in newly diagnosed epilepsy. Neurology. 2007;68(6):402-8.
9. Trinka E, Marson AG, Van Paesschen W, Kalviainen R, Marovac J, Duncan B, et al. KOMET: an unblinded, randomised, two parallel-group, stratified trial comparing the effectiveness of levetiracetam with controlled-release carbamazepine and extended-release sodium valproate as monotherapy in patients with newly diagnosed epilepsy. Journal of neurology, neurosurgery, and psychiatry. 2013;84(10):1138-47.
10. Baulac M, Brodie MJ, Patten A, Segieth J, Giorgi L. Efficacy and tolerability of zonisamide versus controlled-release carbamazepine for newly diagnosed partial epilepsy: a phase 3, randomised, double-blind, non-inferiority trial. Lancet neurology. 2012;11(7):579-88.
11. National Institute for Health and Clinical Excellence. The epilepsies: the diagnosis and management of the epilepsies in adults and children in primary and secondary care.; 2004.
12. Marson AG, Williamson PR, Hutton JL, Clough HE, Chadwick DW. Carbamazepine versus valproate monotherapy for epilepsy. Cochrane database of systematic reviews. 2000(3):CD001030.
13. Tudur Smith C, Marson AG, Williamson PR. Phenytoin versus valproate monotherapy for partial onset seizures and generalized onset tonic-clonic seizures. Cochrane database of systematic reviews. 2001(4):CD001769.
14. Glauser TA, Cnaan A, Shinnar S, Hirtz DG, Dlugos D, Masur D, et al. Ethosuximide, valproic acid, and lamotrigine in childhood absence epilepsy. The New England journal of medicine. 2010;362(9):790-9.
15. Morrow J, Russell A, Guthrie E, Parsons L, Robertson I, Waddell R, et al. Malformation risks of antiepileptic drugs in pregnancy: a prospective study from the UK Epilepsy and Pregnancy Register. Journal of neurology, neurosurgery, and psychiatry. 2006;77(2):193-8.
16. Meador KJ, Baker GA, Browning N, Clayton-Smith J, Combs-Cantrell DT, Cohen M, et al. Cognitive function at 3 years of age after fetal exposure to antiepileptic drugs. The New England journal of medicine. 2009;360(16):1597-605.
17. EMA. New measures to avoid valproate exposure in pregnancy endorsed: Member State representatives agree new restrictions and pregnancy prevention programme. 2018 [cited 2019 21st

For peer review only - http://bmjopen.bmj.com/site/about/guidelines.xhtml

March]. Available from: https://www.ema.europa.eu/en/documents/press-release/new-measures-avoid-valproate-exposure-pregnancy-endorsed_en.pdf.
18. Jacoby A, Baker G, Smith D, Dewey M, Chadwick D. Measuring the impact of epilepsy: the development of a novel scale. Epilepsy research. 1993;16(1):83-8.
19. Ravens-Sieberer U, Bullinger M. Assessing health-related quality of life in chronically ill children with the German KINDL: first psychometric and content analytical results. Quality of life research : an international journal of quality of life aspects of treatment, care and rehabilitation. 1998;7(5):399-407.
20. Cramer JA, Westbrook LE, Devinsky O, Perrine K, Glassman MB, Camfield C. Development of the Quality of Life in Epilepsy Inventory for Adolescents: the QOLIE-AD-48. Epilepsia. 1999;40(8):1114-21.
21. Abetz L, Jacoby A, Baker GA, McNulty P. Patient-based assessments of quality of life in newly diagnosed epilepsy patients: validation of the NEWQOL. Epilepsia. 2000;41(9):1119-28.
22. Beecham J KM. Costing psychiatric interventions. In: G T, editor. Measuring Mental Health Needs. 2nd ed. London: Gaskell; 2001. p. 200-24.
23. NHS. User guide: reference costs 2017/18. 2018 [cited 2019 29th April]. Available from: https://improvement.nhs.uk/documents/1978/6_-_Reference_costs_2017-18_A_Guide_to_using_the_data.pdf.
24. BNF77. BMJ Group and Pharmaceutical Press; 2019.
25. Curtis LA. Unit Costs of Health and Social Care 2018. Personal Social Services Research Unit, University of Kent, Canterbury; 2018.
26. Chadwick D. Considerations on designing clinical trials to evaluate the place of new antiepileptic drugs in the treatment of newly diagnosed and chronic patients with epilepsy. 1998;39(7):799-803.
27. Williamson PR, Smith CT, Sander JW, Marson AG. Importance of competing risks in the analysis of anti-epileptic drug failure. Trials. 2007;8:12.
28. Mauskopf JA, Paul JE, Grant DM, Stergachis A. The role of cost-consequence analysis in healthcare decision-making. PharmacoEconomics. 1998;13(3):277-88.
29. Connock M, Frew E, Evans BW, Bryan S, Cummins C, Fry-Smith A, et al. The clinical effectiveness and cost-effectiveness of newer drugs for children with epilepsy. A systematic review. Health technology assessment. 2006;10(7):iii, ix-118.
30. Wilby J, Kainth A, Hawkins N, Epstein D, McIntosh H, McDaid C, et al. Clinical effectiveness, tolerability and cost-effectiveness of newer drugs for epilepsy in adults: a systematic review and economic evaluation. Health technology assessment. 2005;9(15):1-157, iii-iv.
31. Chan AW, Tetzlaff JM, Altman DG, Laupacis A, Gotzsche PC, Krleza-Jeric K, et al. SPIRIT 2013 statement: defining standard protocol items for clinical trials. Ann Intern Med. 2013;158(3):200-7.
32. EU Directive 2001/20/EC.
33. The Medicines for Human Use (Clinical Trials) Regulations 2004 (SI 2004/1031).

For peer review only - http://bmjopen.bmj.com/site/about/guidelines.xhtml

Reporting checklist for protocol of a clinical trial.

Based on the SPIRIT guidelines.

Instructions to authors

Complete this checklist by entering the page numbers from your manuscript where readers will find each of the items listed below.

Your article may not currently address all the items on the checklist. Please modify your text to include the missing information. If you are certain that an item does not apply, please write "n/a" and provide a short explanation.

Upload your completed checklist as an extra file when you submit to a journal.

In your methods section, say that you used the SPIRITreporting guidelines, and cite them as:

Chan A-W, Tetzlaff JM, Altman DG, Laupacis A, Gøtzsche PC, Krleža-Jerić K, Hróbjartsson A, Mann H, Dickersin K, Berlin J, Doré C, Parulekar W, Summerskill W, Groves T, Schulz K, Sox H, Rockhold FW, Rennie D, Moher D. SPIRIT 2013 Statement: Defining standard protocol items for clinical trials. Ann Intern Med. 2013;158(3):200-207

		Reporting Item	Page Number
**Administrative information**			
Title	#1	Descriptive title identifying the study design, population, interventions, and, if applicable, trial acronym	1

For peer review only

Trial registration	#2a	Trial identifier and registry name. If not yet registered, name of intended registry	3
Trial registration: data set	#2b	All items from the World Health Organization Trial Registration Data Set	3
Protocol version	#3	Date and version identifier	17
Funding	#4	Sources and types of financial, material, and other support	17
Roles and responsibilities: contributorship	#5a	Names, affiliations, and roles of protocol contributors	1-2
Roles and responsibilities: sponsor contact information	#5b	Name and contact information for the trial sponsor	16
Roles and responsibilities: sponsor and funder	#5c	Role of study sponsor and funders, if any, in study design; collection, management, analysis, and interpretation of data; writing of the report; and the decision to submit the report for publication, including whether they will have ultimate authority over any of these activities	15, 16, 17.

For peer review only

Roles and responsibilities: committees	#5d	Composition, roles, and responsibilities of the coordinating centre, steering committee, endpoint adjudication committee, data management team, and other individuals or groups overseeing the trial, if applicable (see Item 21a for data monitoring committee)	15, 16
**Introduction**			
Background and rationale	#6a	Description of research question and justification for undertaking the trial, including summary of relevant studies (published and unpublished) examining benefits and harms for each intervention	4, 5, 6
Background and rationale: choice of comparators	#6b	Explanation for choice of comparators	4, 5, 6
Objectives	#7	Specific objectives or hypotheses	6, 7
Trial design	#8	Description of trial design including type of trial (eg, parallel group, crossover, factorial, single group), allocation ratio, and framework (eg, superiority, equivalence, non-inferiority, exploratory)	7
**Methods: Participants,**			

For peer review only

**interventions, and outcomes**			
Study setting	#9	Description of study settings (eg, community clinic, academic hospital) and list of countries where data will be collected. Reference to where list of study sites can be obtained	7
Eligibility criteria	#10	Inclusion and exclusion criteria for participants. If applicable, eligibility criteria for study centres and individuals who will perform the interventions (eg, surgeons, psychotherapists)	7, 8
Interventions: description	#11a	Interventions for each group with sufficient detail to allow replication, including how and when they will be administered	8, 9, 10
Interventions: modifications	#11b	Criteria for discontinuing or modifying allocated interventions for a given trial participant (eg, drug dose change in response to harms, participant request, or improving / worsening disease)	8, 9,10
Interventions: adherance	#11c	Strategies to improve adherence to intervention protocols, and any procedures for monitoring adherence (eg, drug tablet return; laboratory tests)	8, 9, 10

For peer review only

Interventions: concomitant care	#11d	Relevant concomitant care and interventions that are permitted or prohibited during the trial	9,10
Outcomes	#12	Primary, secondary, and other outcomes, including the specific measurement variable (eg, systolic blood pressure), analysis metric (eg, change from baseline, final value, time to event), method of aggregation (eg, median, proportion), and time point for each outcome. Explanation of the clinical relevance of chosen efficacy and harm outcomes is strongly recommended	6,7, 10, 11, 12,13
Participant timeline	#13	Time schedule of enrolment, interventions (including any run-ins and washouts), assessments, and visits for participants. A schematic diagram is highly recommended (see Figure)	10, 11, 12, 13
Sample size	#14	Estimated number of participants needed to achieve study objectives and how it was determined, including clinical and statistical assumptions supporting any sample size calculations	13,14
Recruitment	#15	Strategies for achieving adequate participant enrolment to reach target sample size	7

For peer review only

Methods: Assignment of interventions (for controlled trials)

Allocation: sequence generation	#16a	Method of generating the allocation sequence (eg, computer-generated random numbers), and list of any factors for stratification. To reduce predictability of a random sequence, details of any planned restriction (eg, blocking) should be provided in a separate document that is unavailable to those who enrol participants or assign interventions	8
Allocation concealment mechanism	#16b	Mechanism of implementing the allocation sequence (eg, central telephone; sequentially numbered, opaque, sealed envelopes), describing any steps to conceal the sequence until interventions are assigned	8
Allocation: implementation	#16c	Who will generate the allocation sequence, who will enrol participants, and who will assign participants to interventions	8
Blinding (masking)	#17a	Who will be blinded after assignment to interventions (eg, trial participants, care	n/a

For peer review only

		providers, outcome assessors, data analysts), and how	
Blinding (masking): emergency unblinding	#17b	If blinded, circumstances under which unblinding is permissible, and procedure for revealing a participant's allocated intervention during the trial	n/a
**Methods: Data collection, management, and analysis**			
Data collection plan	#18a	Plans for assessment and collection of outcome, baseline, and other trial data, including any related processes to promote data quality (eg, duplicate measurements, training of assessors) and a description of study instruments (eg, questionnaires, laboratory tests) along with their reliability and validity, if known. Reference to where data collection forms can be found, if not in the protocol	15,16
Data collection plan: retention	#18b	Plans to promote participant retention and complete follow-up, including list of any outcome data to be collected for participants who discontinue or deviate from intervention protocols	15,16

For peer review only

Data management	#19	Plans for data entry, coding, security, and storage, including any related processes to promote data quality (eg, double data entry; range checks for data values). Reference to where details of data management procedures can be found, if not in the protocol	16
Statistics: outcomes	#20a	Statistical methods for analysing primary and secondary outcomes. Reference to where other details of the statistical analysis plan can be found, if not in the protocol	14
Statistics: additional analyses	#20b	Methods for any additional analyses (eg, subgroup and adjusted analyses)	14,15
Statistics: analysis population and missing data	#20c	Definition of analysis population relating to protocol non-adherence (eg, as randomised analysis), and any statistical methods to handle missing data (eg, multiple imputation)	14,15
**Methods: Monitoring**			
Data monitoring: formal committee	#21a	Composition of data monitoring committee (DMC); summary of its role and reporting structure; statement of whether it is independent from the sponsor and competing interests; and reference to where further	15, 16

For peer review only

		details about its charter can be found, if not in the protocol. Alternatively, an explanation of why a DMC is not needed	
Data monitoring: interim analysis	#21b	Description of any interim analyses and stopping guidelines, including who will have access to these interim results and make the final decision to terminate the trial	14
Harms	#22	Plans for collecting, assessing, reporting, and managing solicited and spontaneously reported adverse events and other unintended effects of trial interventions or trial conduct	14,16
Auditing	#23	Frequency and procedures for auditing trial conduct, if any, and whether the process will be independent from investigators and the sponsor	N/A
**Ethics and dissemination**			
Research ethics approval	#24	Plans for seeking research ethics committee / institutional review board (REC / IRB) approval	15, 17
Protocol amendments	#25	Plans for communicating important protocol modifications (eg, changes to eligibility criteria, outcomes, analyses) to relevant	15

For peer review only

		parties (eg, investigators, REC / IRBs, trial participants, trial registries, journals, regulators)	
Consent or assent	#26a	Who will obtain informed consent or assent from potential trial participants or authorised surrogates, and how (see Item 32)	7, 15
Consent or assent: ancillary studies	#26b	Additional consent provisions for collection and use of participant data and biological specimens in ancillary studies, if applicable	12-13
Confidentiality	#27	How personal information about potential and enrolled participants will be collected, shared, and maintained in order to protect confidentiality before, during, and after the trial	17
Declaration of interests	#28	Financial and other competing interests for principal investigators for the overall trial and each study site	17
Data access	#29	Statement of who will have access to the final trial dataset, and disclosure of contractual agreements that limit such access for investigators	17
Ancillary and post trial care	#30	Provisions, if any, for ancillary and post-trial care, and for compensation to those who suffer harm from trial participation	8-10

For peer review only

Dissemination policy: trial results	#31a	Plans for investigators and sponsor to communicate trial results to participants, healthcare professionals, the public, and other relevant groups (eg, via publication, reporting in results databases, or other data sharing arrangements), including any publication restrictions	17
Dissemination policy: authorship	#31b	Authorship eligibility guidelines and any intended use of professional writers	15
Dissemination policy: reproducible research	#31c	Plans, if any, for granting public access to the full protocol, participant-level dataset, and statistical code	17
**Appendices**			
Informed consent materials	#32	Model consent form and other related documentation given to participants and authorised surrogates	Available from the trial website www.sanad2.org.uk .
Biological specimens	#33	Plans for collection, laboratory evaluation, and storage of biological specimens for genetic or molecular analysis in the current trial and for future use in ancillary studies, if applicable	12-13

None The SPIRIT checklist is distributed under the terms of the Creative Commons Attribution License CC-BY-ND 3.0. This checklist can be completed online using https://www.goodreports.org/, a tool made by the EQUATOR Network in collaboration with Penelope.ai

For peer review only

BMJ Open

A study protocol for a pragmatic randomised controlled trial comparing the effectiveness and cost effectiveness of levetiracetam and zonisamide versus standard treatments for epilepsy: a comparison of Standard And New Antiepileptic Drugs (SANAD-II).

Journal:	*BMJ Open*
Manuscript ID	bmjopen-2020-040635.R1
Article Type:	Protocol
Date Submitted by the Author:	18-Jul-2020
Complete List of Authors:	Balabanova, Silviya; University of Liverpool Faculty of Health and Life Sciences, Liverpool Clinical Trials Centre<br>Taylor, Claire; University of Liverpool Faculty of Health and Life Sciences, Liverpool Clinical Trials Centre<br>Sills, Graeme; University of Glasgow, School of Life Sciences<br>Burnside, Girvan; University of Liverpool Faculty of Health and Life Sciences, Biostatistics<br>Plumpton, Catrin; Bangor University, Centre for Health Economics and Medicines Evaluation<br>Smith, Phil; University Hopital of Wales, Department of Neurology<br>Appleton, Richard; Alder Hey Children's NHS Foundation Trust, Neurology<br>Leach, John; University of Glasgow, School of Medicine<br>Johnson, Michael; Imperial College London Faculty of Medicine - South Kensington Campus<br>Baker, Gus; University of Liverpool Faculty of Health and Life Sciences, Molecular and Clinical Pharmacology<br>Pirmohamed, Munir; University of Liverpool Faculty of Health and Life Sciences, Department of Pharmacology<br>Hughes, Dyfrig; Bangor University, Centre for Health Economics and Medicines Evaluation<br>Williamson, Paula; University of Liverpool, Biostatistics<br>Tudur-Smith, Catrin; University of Liverpool, Biostatistics<br>Marson, Anthony; University of Liverpool, Molecular and Clinical Pharmacology
**Primary Subject Heading**:	Neurology
Secondary Subject Heading:	Health policy, Health economics
Keywords:	Epilepsy < NEUROLOGY, Health policy < HEALTH SERVICES ADMINISTRATION & MANAGEMENT, Health economics < HEALTH SERVICES ADMINISTRATION & MANAGEMENT, Paediatric neurology < PAEDIATRICS, Clinical trials < THERAPEUTICS

For peer review only - http://bmjopen.bmj.com/site/about/guidelines.xhtml

For peer review only - http://bmjopen.bmj.com/site/about/guidelines.xhtml

I, the Submitting Author has the right to grant and does grant on behalf of all authors of the Work (as defined in the below author licence), an exclusive licence and/or a non-exclusive licence for contributions from authors who are: i) UK Crown employees; ii) where BMJ has agreed a CC-BY licence shall apply, and/or iii) in accordance with the terms applicable for US Federal Government officers or employees acting as part of their official duties; on a worldwide, perpetual, irrevocable, royalty-free basis to BMJ Publishing Group Ltd ("BMJ") its licensees and where the relevant Journal is co-owned by BMJ to the co-owners of the Journal, to publish the Work in this journal and any other BMJ products and to exploit all rights, as set out in our licence.

The Submitting Author accepts and understands that any supply made under these terms is made by BMJ to the Submitting Author unless you are acting as an employee on behalf of your employer or a postgraduate student of an affiliated institution which is paying any applicable article publishing charge ("APC") for Open Access articles. Where the Submitting Author wishes to make the Work available on an Open Access basis (and intends to pay the relevant APC), the terms of reuse of such Open Access shall be governed by a Creative Commons licence – details of these licences and which Creative Commons licence will apply to this Work are set out in our licence referred to above.

Other than as permitted in any relevant BMJ Author's Self Archiving Policies, I confirm this Work has not been accepted for publication elsewhere, is not being considered for publication elsewhere and does not duplicate material already published. I confirm all authors consent to publication of this Work and authorise the granting of this licence.

For peer review only

TITLE PAGE

**Title**	**A study protocol for a pragmatic randomised controlled trial comparing the effectiveness and cost effectiveness of levetiracetam and zonisamide versus standard treatments for epilepsy: a comparison of Standard And New Antiepileptic Drugs (SANAD-II)**
**Authors' names**	Silviya Balabanova (0000-0001-6989-8099), Claire Taylor, Graeme Sills (0000-0002-3389-8713), Girvan Burnside, Catrin Plumpton, Phil Smith, Richard Appleton (0000-0002-0742-2113), John Paul Leach (0000-0003-2086-9937), Michael Johnson, Gus Baker, Munir Pirmohamed, Dyfrig Hughes (0000-0001-8247-7459), Paula Williamson (0000-0001-9802-6636), Catrin Tudur-Smith, Anthony Marson (0000-0002-6861-8806)
**Address for each author**<br>Separate each author's name with a comma (don't use a separate line for each part) Please include postcodes<br>**Authors' names and positions**<br>Use same style of name as below title Please give one job position for each author (under the relevant address) on separate line from author's name	Clinical Trials Research Centre University of Liverpool 2nd Floor – Institute in the Park Alder Hey Children's NHS Foundation Trust Eaton Road Liverpool L12 2AP Silviya Balabanova, clinical trial co-ordinator<br>Clinical Trials Research Centre University of Liverpool 2nd Floor – Institute in the Park Alder Hey Children's NHS Foundation Trust Eaton Road Liverpool L12 2AP Claire Taylor, clinical trial co-ordinator<br>School of Life Sciences Room 346 Sir James Black Building University of Glasgow Glasgow G12 8QQ Graeme Sills, Lecturer in Pharmacology<br>Department of Biostatistics Block F Waterhouse Building University of Liverpool 1-5 Brownlow Street Liverpool L69 3GL Girvan Burnside, Senior lecturer<br>Centre for Health Economics and Medicines Evaluation Ardudwy Bangor University Holyhead Road Bangor Wales LL57 2PZ Catrin Plumpton, Research Fellow<br>The Alan Richens Epilepsy Unit University Hospital of Wales Heath Park Cardiff CF14 4XW Phil Smith, Consultant Neurologist<br>The Roald Dahl EEG Unit Alder Hey Children's Health Park Liverpool L12 2AP Richard Appleton, Consultant Paediatric Neurologist<br>School of Medicine Wolfson Medical School Building University Avenue University of Glasgow G12 8QQ John Paul Leach, Head of Undergraduate Medicine<br>Division of Brain Science Imperial College London SW7 2AZ Michael Johnson, Professor of Neurology and Genomic Medicine<br>Department of Molecular and Clinical Pharmacology University of Liverpool Clinical Sciences Centre Lower Lane Liverpool L9 7LJ Gus Baker, Professor of Clinical Neuropsychology<br>Department of Molecular and Clinical Pharmacology University of Liverpool Ashton Street Liverpool L69 3GE Munir Pirmohamed, Professor of Molecular and Clinical Pharmacology<br>Centre for Health Economics and Medicines Evaluation Ardudwy Bangor University Holyhead Road Bangor Wales LL57 2PZ Dyfrig Hughes, Professor of Health Economics<br>Department of Biostatistics Block F Waterhouse Building University of Liverpool 1-5 Brownlow Street Liverpool L69 3GL Paula Williamson, Professor of Biostatistics

For peer review only

For peer review only - http://bmjopen.bmj.com/site/about/guidelines.xhtml

	Department of Biostatistics Block F Waterhouse Building University of Liverpool 1-5 Brownlow Street Liverpool L69 3GL Catrin Tudur-Smith, Professor of Biostatistics<br>Department of Molecular and Clinical Pharmacology University of Liverpool Clinical Sciences Centre Lower Lane Liverpool L9 7LJ Anthony Guy Marson, Professor of Neurology
**Corresponding author**	Anthony Guy Marson<br>Professor of Neurology<br>Email to: marjon01@liverpool.ac.uk<br>Tel: 0151 529 5770
**Word count**	4,524

For peer review only

Keywords: epilepsy, SANAD-II, SANAD-I, antiepileptic drugs (AED), sodium valproate, levetiracetam, lamotrigine, zonisamide, treatment policy, effectiveness, cost effectiveness, health economics.

ABSTRACT

Introduction: Antiepileptic drugs (AEDs) are the mainstay of epilepsy treatment. Over the past 20 years, a number of new drugs have been approved for NHS use on the basis of information from short term trials that demonstrate efficacy. These trials do not provide information about the longer term outcomes, which inform treatment policy. This trial will assess the long term clinical and cost-effectiveness of the newer treatments levetiracetam and zonisamide.

Methods and analysis: This is a phase IV, multicentre, open-label, randomised, controlled clinical trial comparing new and standard treatments for patients with newly-diagnosed epilepsy. Arm A of the trial randomised 990 patients with focal epilepsy to standard AED lamotrigine or new AEDs levetiracetam or zonisamide. Arm B randomised 520 patients with generalised epilepsy to standard AED sodium valproate or new AED levetiracetam. Patients are recruited from United Kingdom NHS out-patient epilepsy, general neurology and paediatric clinics. Included patients are aged 5 years or older with two or more spontaneous seizures requiring AED monotherapy, who are not previously treated with AEDs. Patients are followed-up for a minimum of 2 years. The primary outcome is time to 12-month remission from seizures. Secondary outcomes include time to treatment failure (including due to inadequate seizure control or due to unacceptable adverse reactions); time to first seizure; time to 24-month remission; adverse reactions and quality of life. All primary analyses will be on an intention to treat basis. Separate analyses will be undertaken for each arm. Health economic analysis will be conducted from the perspective of the NHS to assess the cost-effectiveness of each AED.

For peer review only - http://bmjopen.bmj.com/site/about/guidelines.xhtml

Ethics and dissemination: This trial has been approved by the North West-Liverpool East REC (Ref. 12/NW/0361). The trial team will disseminate the results through scientific meetings, peer reviewed publications and patient and public involvement.

Trial registration: EudraCT 2012-001884-64; ISRCTN30294119.

ARTICLE SUMMARY

Strengths and limitations of this study

- The study is adequately powered to examine the clinical effectiveness of standard and new anti-epileptic drugs (AEDs)
- The study is adequately powered to examine the cost effectiveness of standard and AEDS
- This is a long term trial which will provide evidence of the long term effects of some AEDs

- A limitation is that the study is not blinded.
- A further limitation is the reliance on patient completed questionnaires, which should be offset by access to hospital episode statistic data.

INTRODUCTION

Epilepsy is a common neurological condition and up to 3% of people will experience seizures at some time in their lives (1). Epilepsy is a complex condition with many different causes and seizures can take many different forms. It is uniquely stigmatising and has a negative impact on quality of life and employment prospects (2, 3). Antiepileptic drugs (AEDs) are the mainstay of treatment and may have to be taken for life. The ultimate goal of treatment is to maximise quality of life by eliminating seizures at drug doses that do not cause side effects. However, for many patients there is a necessary trade-off between effective seizure control and side effects, which can diminish quality of life.

Over the past 20 years, a number of new drugs have become available for the treatment of epilepsy. These new drugs have been approved for NHS use on the basis of information from short term trials. These trials do not provide information about the longer term outcomes which inform decisions made by doctors and patients, nor do they provide any useful health economic data to inform policy.

For peer review only - http://bmjopen.bmj.com/site/about/guidelines.xhtml

For peer review only

The SANAD-I trial began in 1999 and compared the effectiveness and cost effectiveness of standard and new treatments that were available at that time (4, 5). SANAD-I identified lamotrigine (a new drug) as an effective and cost-effective first-line treatment for patients with a focal epilepsy, and confirmed that valproate (a standard treatment) should remain a first-line drug for patients with a generalised epilepsy or seizures that clinicians find difficult to classify. Since SANAD-I, a number of newer treatments have become available, the most promising of which are levetiracetam and zonisamide. SANAD-II will include two randomised controlled trials (RCTs) run in parallel; Arm A recruiting patients with focal epilepsy and Arm B patients with generalised or unclassified epilepsy.

Rationale

Arm A

Arm A (focal epilepsy) of SANAD-II will compare lamotrigine, levetiracetam and zonisamide in patients with untreated focal onset seizures. While the focal epilepsies are further classified into a number of syndromes (6) largely according to aetiology and site of onset, it has been common practice to recruit a heterogeneous population with focal onset seizures into epilepsy trials. There are currently no reliable data that indicate whether relative treatment responses differ among the focal epilepsies, indeed prognostic modelling of data from SANAD-I suggests that treatment effects are consistent across focal epilepsy syndromes as currently classified (4, 7). In SANAD-II, we will recruit patients with focal onset seizures irrespective of syndrome, which will allow further opportunity to investigate treatment effects and factors, including syndrome, that influence those effects.

Lamotrigine was chosen as the 'standard' treatment as in SANAD-I it was found to be superior to carbamazepine for time to treatment failure and non-inferior to carbamazepine for time to 12 month remission, whilst the health economic analysis found lamotrigine a cost effective alternative to carbamazepine (4).

Levetiracetam is a commonly used antiepileptic drug with evidence for efficacy (non-inferiority to carbamazepine for 6 month seizure remission) as monotherapy in focal epilepsy from regulatory studies with too short a duration of follow-up to inform policy (8). A second industry-sponsored un-blinded trial compared levetiracetam with physicians' choice of carbamazepine or valproate, but again follow-up was too short to inform treatment policy (9).

Zonisamide is a drug that has been available for many years in Japan and other countries in South East Asia where it is commonly used both as initial monotherapy and as an add-on treatment, and is licensed for use in the EU and USA. Evidence for efficacy is from industry sponsored regulatory

For peer review only - http://bmjopen.bmj.com/site/about/guidelines.xhtml

studies demonstrating non-inferiority when compared with carbamazepine for 6 month seizure remission rates (10).

Arm B

Arm B (generalised or unclassified epilepsy) of SANAD-II will compare levetiracetam and valproate in patients with generalised onset seizures or seizures that are difficult to classify. Generalised onset seizures represent a group of syndromes, most of which are currently classified as one of the idiopathic generalised epilepsies (6), which are largely classified according to seizure type and age of onset. While differing syndromes are recognised, there is currently no reliable evidence that relative treatment responses differ among syndromes, indeed prognostic modelling of data from SANAD-I indicates that relative treatment responses are consistent across syndromes. As in SANAD-I, patients enter Arm B of the trial based upon a classification of seizures (generalised onset or difficult to classify), with patients further classified by syndrome where and when such a syndromic diagnosis can be made.

Few randomised controlled trials have been undertaken to assess the comparative effects of antiepileptic drugs in patients with generalised onset seizures, or in those with seizures that are difficult to classify, even though these individuals represent over one third of people with epilepsy. Valproate has for some time been recommended as a first line treatment for such patients (11), but without evidence from RCTs to support this recommendation. Cochrane reviews have compared valproate with other antiepileptic drugs (12, 13), but due to problems with power and epilepsy classification, none have showed an advantage for valproate. In Arm B of SANAD-I (5), valproate was significantly more effective than lamotrigine and significantly better tolerated than topiramate. Also, a double blind trial of 16 weeks therapy in childhood and juvenile absence epilepsy (14), valproate and ethosuximide were significantly superior to lamotrigine for the outcome treatment failure. Valproate however remains a difficult drug for women of childbearing potential as it is associated with a higher rate of teratogenicity than alternatives (major malformation rate ~8%) (15). There is also evidence that valproate can affect the intellectual development of children exposed in utero with up to one third of children having a significant reduction in their IQ (16). In 2018, the EMA and MHRA launched a pregnancy prevention programme for women taking valproate, and state that women of childbearing potential should not be prescribed valproate unless other treatments are ineffective or not tolerated (17). For women with generalised epilepsy, making a treatment choice is very challenging, with options including valproate, lamotrigine which is less effective but safer in pregnancy, or levetiracetam for which the efficacy when compared to valproate is unknown and for which we have increasing evidence of relative safety in pregnancy.

For peer review only - http://bmjopen.bmj.com/site/about/guidelines.xhtml

TRIAL OUTCOMES

Primary Outcome

The primary outcome of the trial is time to 12-month remission from seizures.

Secondary outcomes

The secondary outcomes are:

a. Time to treatment failure
b. Time to treatment failure due to inadequate seizure control. This event will have occurred when the clinician and/or patient decide that treatment replacement or withdrawal, or the addition of a second antiepileptic drug is required due to the occurrence of a seizure on the maximum recommended dose of randomised drug, or the maximum tolerated dose of the drug.
c. Time to treatment failure due to unacceptable adverse events. This event will have occurred when the patient experiences adverse events attributed to the drug necessitating its withdrawal.
d. Time to first seizure
e. Time to 24-month remission
f. Adverse reactions (ARs)
g. QOL
h. Health economic outcomes expressed as the incremental cost per Quality-Adjusted Life-Year (QALY) gained

TRIAL DESIGN

SANAD-II trial is a phase IV, multicentre, randomised, open-label controlled trial. Arm A of the trial recruits participants diagnosed with focal epilepsy and Arm B - with generalised or unclassified epilepsy. Trial participants are randomised to an antiepileptic drug (AED) within each Arm. In Arm A the control treatment is lamotrigine and the new treatments are levetiracetam and zonisamide, in treatment group ratio of 1:1:1. In Arm B the new treatment levetiracetam is compared to the control treatment valproate in treatment group ratio of 1:1. The sample size for the trial is 1,510 participants (990 with focal epilepsy and 520 with generalised or unclassified epilepsy).

ENROLMENT AND RANDOMISATION

Screening and baseline

The SANAD-II trial will take place in NHS out-patient epilepsy, general neurology and paediatric (epilepsy and general) clinics in the UK (a full list of participating sites is available from the trial

For peer review only - http://bmjopen.bmj.com/site/about/guidelines.xhtml

website). All patients meeting the eligibility criteria (Table 1) will be invited to participate in the study and provided with an age and development-appropriate patient information sheet and consent form. Patients will be allowed sufficient time to discuss trial and to decide on their participation. Once consent has been obtained the baseline data will be collected and patient will be progressed to randomisation.

**Table 1. Eligibility Criteria**	
Inclusion Criteria	a. Aged 5 years or older<br>b. Two or more spontaneous seizures that require antiepileptic drug treatment<br>c. Untreated and not previously treated with antiepileptic drugs, except emergency treatment in the past 2 weeks<br>d. Antiepileptic drug monotherapy considered the most appropriate option<br>e. Willing to provide consent (patients parent/legal representative willing to give consent where the patient is aged under 16 years of age or is lacking capacity to consent)
Exclusion Criteria	a. Provoked seizures only (e.g. alcohol or drug-induced)<br>b. Acute symptomatic seizures only (e.g. within 1 month from acute brain haemorrhage or brain injury or stroke)<br>c. Currently treated with antiepileptic drugs<br>d. Progressive neurological disease (e.g. known brain tumour)

Randomisation

The arm (A or B) to which the patient is assigned will be decided by the recruiting physician based upon their epilepsy classification. Patients will then be randomised to one of the following treatments: to lamotrigine, levetiracetam or zonisamide (in Arm A) or to levetiracetam or valproate (in Arm B). Randomisation will use a minimisation program with a built in random element utilising factors that will not be made known to individuals in charge of recruitment to minimise any potential for predicting allocation. Participants will be randomised using a secure (24-hour) web based randomisation programme controlled centrally by the Clinical Trials Research Centre (CTRC). Randomised treatment should begin within 7 days of randomisation. The research team should ensure that the duration between obtaining consent, performing baseline assessments, randomisation and the start of trial treatment does not impact on the well-being of the participant.

For peer review only - http://bmjopen.bmj.com/site/about/guidelines.xhtml

TRIAL INTERVENTIONS

SANAD-II is a pragmatic trial that uses market authorised drugs within the terms of marketing authorisation. All treatments will be taken as formulations already licensed to be used in UK and there will be no modifications made to the products or their outer packaging. All treatments will be prescribed as per routine NHS practice and dispensed by hospital and community pharmacies as they would normally be.

It is accepted that, for a variety of reasons including perceived or actual efficacy and tolerability, not all patients will take their medicines as prescribed. Patients will be asked about adherence in the QoL questionnaires, but no objective measurements of adherence are planned nor will the primary analyses be adjusted for actual or estimated adherence.

SANAD-II is an unblinded trial therefore decisions about concomitant medications/treatments will depend on the local medical plan and clinical management.

All patients will be titrated to an initial maintenance dose, with dose adjustments made at subsequent appointments according to the clinical response and adverse effects. Guidelines for titration and initial maintenance dose are provided within the protocol (Table 2), however clinicians will be able to alter this to choose the titration rate and initial maintenance they think most appropriate for individual patients.

**Table 2. Titration and Initial Maintenance Dose**		
**Arm A**		
Age > 12 years.		
**Lamotrigine**	**Levetiracetam**	**Zonisamide**
25mg once per day for 2 weeks<br>25mg twice per day for 2 weeks<br>50mg twice per day for 2 weeks<br>50mg morning and 100mg at night - *initial target maintenance dose*	250mg once per day for 2 weeks<br>250mg twice per day for 2 weeks<br>250mg morning and 500mg night for 2 weeks<br>500mg twice per day - *initial target maintenance dose*	50mg once per day 2 weeks<br>50mg twice per day for 2 weeks<br>50mg am 100mg pm for 2 weeks<br>100mg am 100mg pm - *initial target maintenance dose*
Children aged 5-12 years.		
**Lamotrigine**	**Levetiracetam**	**Zonisamide**
0.5 mg/kg/ day as a once a day dose for 2 weeks<br>0.5 mg/kg/day as a twice daily regimen for 2 weeks<br>0.5 mg/kg am and 1.0 mg/kg pm for 2 weeks	10 mg/kg/day as a twice daily regimen for 2 weeks<br>20 mg/kg/day as a twice daily regimen for 2 weeks<br>30 mg/kg/day as a twice daily regimen for 2 weeks	0.5 - 1 mg/kg/day as a once or twice daily regimen (depending on the child's weight) for 2 weeks<br>1 - 1.5 (maximum) mg/kg/day as a twice daily regimen for 2 weeks<br>2 - 2.5 (maximum) mg/kg/day as a twice daily regimen for 2 weeks<br>3 - 4 mg/kg/day as a twice daily

For peer review only - http://bmjopen.bmj.com/site/about/guidelines.xhtml

1.0 mg/kg am and 1.0 mg/kg pm for 2 weeks<br>1.5 mg/kg am and 1.5 mg/kg pm – *initial target maintenance dose*	40 mg/kg/day as a twice daily regimen – *initial target maintenance dose*	regimen for 2 weeks<br>5 mg/kg/day as a twice daily regimen - *initial target maintenance dose*.

**Arm B**	
Age > 12 years.	
**Valproate**<br>500mg once per day for 2 weeks<br>500mg twice per day - *Initial target maintenance dose*	**Levetiracetam**<br>250mg once per day for 2 weeks<br>250mg twice per day for 2 weeks<br>250mg morning and 500mg night for 2 weeks<br>500mg twice per day - *initial target maintenance dose*
Children aged 5-12 years.	
**Valproate**<br>10 mg/kg/day as a twice daily regimen for 2 weeks<br>15 mg/kg/day as a twice daily regimen for 2 weeks<br>25 mg/kg/day as a twice daily regimen – *initial target maintenance dose*	**Levetiracetam**<br>10 mg/kg/day as a twice daily regimen for 2 weeks<br>20 mg/kg/day as a twice daily regimen for 2 weeks<br>30 mg/kg/day as a twice daily regimen for 2 weeks<br>40 mg/kg/day as a twice daily regimen – *initial target maintenance dose*

For peer review only

The aim of treatment will be to control seizures with a minimum effective dose of drug. This will necessitate dosage modification (dose increased or reduced) if further seizures or adverse events occur as is usual clinical practice. Any changes in medication must be documented along with the justification for those changes. At the end of trial participation the participants may continue their treatment as per local policy.

To avoid potentially confounding issues, ideally patients should not be recruited into other epilepsy trials.

ASSESSMENTS AND PROCEDURES

Schedule for Follow up

Patients were recruited over a 4.5 year period and follow-up will continue for a further two years. The maximum time that a patient will receive their randomised treatment is 6.5 years. Table 3 shows the schedule of follow-up.

All participants will be followed up whether they are still taking their allocated treatment or not. Where patients default from clinic follow-up, additional information will be sought from General Practitioners (GP) who will be the main prescribers of AEDs in this trial. Patients will be followed up as per routine clinical practice and typically at 3, 6, and 12 months and annually thereafter. Patients may be seen at other times as clinically indicated.

For peer review only - http://bmjopen.bmj.com/site/about/guidelines.xhtml

Where treatment is stopped the participant will be asked to continue with the trial follow-up and to attend the follow-up visits.

Table 3 – Schedule of follow-up

Procedures	Baseline (T0)[1]	Follow Up Schedule: T0+3 months	T0+6 months	T0+12 months and annually thereafter
Signed Consent Form	X			
Assessment of Eligibility Criteria	X			
Contact details	X			
Review of Medical History including:<br>• Seizure history<br>• Neurological insult<br>• Febrile seizures<br>• Family history of epilepsy<br>• EEG results<br>• Imaging results (CT or MRI)	X			
Further investigation (EEG/ CT/ MRI)	(X)			
Allocation of Study Treatment	X			
Issue of Questionnaires in person or by post	X	X	X	X
Review of seizure occurrence & hospital admissions		X	X	X
Review of AED use (Study Treatment & Concomitant):<br>• Since last follow up<br>• Changes made to treatment plan including reasons		X	X	X
Assessment of Adverse Reactions		(X)	(X)	(X)
Resource use		X	X	X
Re-Issue of Questionnaire by post or at site to non-responders typically three weeks later	(X)	(X)	(X)	(X)
Telephone follow-up of questionnaire non-responders typically three weeks later	(X)	(X)	(X)	(X)
Special Assay or Procedure: Consent and obtain saliva or blood sample for later DNA analysis	(X)			

(X) – As indicated/appropriate.
1 At baseline, all procedures should be done before study intervention.

Efficacy of the trial treatments will be measured throughout the trial using a number of measures. Data on seizures will provide a subjective measure of efficacy. Quality of life data obtained

For peer review only - http://bmjopen.bmj.com/site/about/guidelines.xhtml

throughout the trial using age appropriate questionnaire booklets (Table 4) can be used as a subjective measure of efficacy.

Assessment of ARs will be undertaken at each study visit.

Quality of Life (QoL) and Utility assessments

For adults, QOL outcomes will be assessed using subscales of the NEWQOL battery and the Impact of Epilepsy Scale (18). For children and adolescents aged < 16 years, QOL assessment will involve both patient and parent-based measures: children aged 8-15 years will complete a generic health status measure validated for use in epilepsy, the KINDL (19); and the 'epilepsy impact' and 'attitude to epilepsy' subscales of the QOLIE-AD (20). Parents of all children will also complete proxy QOL questionnaires.

Utility scores will be elicited directly from trial participants (or indirectly via parents / guardians). Adult and adolescent patients will be asked to complete the EQ-5D-3L questionnaire and Visual Analogue Scale (VAS). The EQ-5D-3L has been used previously in children, but it has not been formally validated (21), and EQ-5D-3L weights are validated for adults aged ≥18 years. The currently recommended approach of using parental proxy reports of quality of life for this age group will be used (22). EQ-5D-3L-Y (youth version) will additionally be administered to children aged 8-15 years. All trial participants will be asked to complete an epilepsy-specific utility measure, based on the NEWQOL-6D questionnaire (23).

Patients and/or parents/guardians will be completing questionnaires at baseline and at specific time points throughout the trial.

Table 4: Age-appropriate questionnaires booklets

Participant age	Questionnaire booklet completed by	Questionnaire booklet content
5 - 7	Participant	N/A
	Parent/Carer	Kiddy-KINDL, EQ-5D-3L & EQ-VAS, NEWQOL-6D
8 - 11 (Children)	Participant	Kid-KINDL, EQ-5D-3L-Y & EQ-VAS, QOLIE-AD
	Parent/Carer	Kid-KINDL, EQ-5D-3L & EQ-VAS, NEWQOL-6D
12 - 15 (Young people)	Participant	Kiddo-KINDL, EQ-5D-3L-Y & EQ-VAS, QOLIE-AD
	Parent/Carer	Kiddo-KINDL, EQ-5D-3L & EQ-VAS, NEWQOL-6D
≥ 16 (Adult)	Participant	Impact of Epilepsy Scale, EQ-5D-3L & EQ-VAS, NEWQOL-6D

Resources Use and Cost Data

Direct costs of health care resources used by patients in the trial will be collected in three ways:

For peer review only - http://bmjopen.bmj.com/site/about/guidelines.xhtml

i. A modified version of the Client Service Receipt Inventory (CSRI) (24) to assess patients' use of primary and community care services and personal social services (e.g. primary care services (NHS Direct, Walk-in treatment centres, and home visits etc.).

ii. Patients' use of secondary care services as Hospital Episode Statistics (HES) data. Downloaded Healthcare Resource Group (HRG) data will include information on out-patient epilepsy, general neurology and paediatric clinics visits; accident and emergency attendance, and length (and nature) of hospitalisations.

iii. Resources triggered by ARs will be captured in the follow-up CRF for each patient experiencing a serious AR requiring hospitalisation. Because of potential issues related to completeness of routine data, these will be used to compliment HES data.

Unit costs will be taken from the NHS reference costs database (25); and other appropriate sources (26, 27).

Genetic sub-study

DNA collection will be included as an option in SANAD-II in order that refusal will not preclude from trial participation. Whole blood or saliva samples will be shipped to a central laboratory at The University of Liverpool for extraction and storage. No genotyping of DNA samples will be undertaken as part of SANAD-II. Samples will instead be genotyped in future projects and data arising from that analysis included in international epilepsy genomics initiatives. Identification of genetic factors associated with response to treatment in epilepsy is important and may ultimately help to optimise efficacy, tolerability and safety of antiepileptic drugs.

SAMPLE SIZE CALCULATION

SANAD-II is powered to detect non-inferiority (NI) of the new antiepileptic drugs (levetiracetam and zonisamide) compared to standard treatments (lamotrigine or valproate) for the primary outcome time to 12-month remission. A new drug might become a standard first line treatment if it is proven to be non-inferior for efficacy but superior for tolerability when compared to a standard treatment – tolerability is examined in secondary outcomes including time to treatment failure for adverse effects. Powering the study for non-inferiority will also provide sufficient power to detect important differences between treatment policies.

The ILAE Commission on Antiepileptic Drugs defined limits of equivalence of +/- 10% for the primary outcome in antiepileptic drug monotherapy studies (28). However the Commission was not explicit as to whether this should be on the hazard ratio (HR) or absolute scale. No empirical work has yet been undertaken to underpin the choice of equivalence or non-inferiority margins in epilepsy trials.

For peer review only - http://bmjopen.bmj.com/site/about/guidelines.xhtml

The Chief Investigator has given numerous seminars and lectures in the UK and elsewhere about epilepsy trial methodology and the audience typically vote for a margin of 10% around absolute differences between AEDs for monotherapy studies when given examples of margins ranging from 20% to 5%.

Calculations have been informed by the SANAD-I study which estimated the 12 month remission-free probability (at 24 months) as 0.43 (exponential hazard rate of 0.0352) for lamotrigine (Arm A standard) and 0.31 (exponential hazard rate of 0.0488) for valproate (Arm B standard). The calculations assume a HR of 1.0, 80% power, and allowance for approximately 5% losses to follow-up throughout, as occurred in SANAD-I. For patients with focal-onset seizures (Arm A) two primary comparisons are of interest (levetiracetam vs lamotrigine and zonisamide vs lamotrigine), therefore the one-sided significance level has been divided by two (one-side alpha 0.0125). Assuming a 10% absolute difference in survival probability, the non-inferiority margin on the HR scale is ln(0.43)/ln(0.53)=1.329. After adjusting for 5% losses to follow-up, 330 patients are required in each of 3 treatment groups (990 total for Arm A). For patients with generalised-onset seizures or seizures that are difficult to classify (Arm B) there is only one comparison of interest (levetiracetam vs valproate). Assuming a 10% absolute difference in survival probability, the non-inferiority margin on the HR scale is ln(0.31)/ln(0.41)=1.314 for Arm B. Therefore, with a one-sided alpha of 0.025, 260 patients are required in each of two treatment groups allowing for 5% losses to follow-up (520 total for Arm B). The total number of patients required is 1510.

STATISTICAL ANALYSIS

All primary analyses will be on an intention to treat (ITT) basis including all randomised patients retained in their randomised treatment groups. Separate analyses will be undertaken for each randomisation arm. The interval (in days) from randomisation to occurrence of a 12-month remission will be summarised by Kaplan-Meier curves for each treatment group. Survival regression models will be explored; two different models will be used: (i) including the treatment effect only using treatment indicator variables; (ii) including the treatment effect together with covariates. The impact of centre effect on the treatment comparison will be investigated using both fixed and random effect models. A per protocol analysis will be undertaken to assess the robustness of ITT analyses. For Arm B trial, an additional analysis of the primary outcome will add a stratification variable to the model: seizure type (generalised / unclassified).

A similar analysis strategy will be employed for the other secondary time to event outcomes. For time to treatment failure, further analysis will be undertaken to assess the two main reasons for treatment failure - inadequate seizure control and unacceptable adverse effects. To allow for

For peer review only - http://bmjopen.bmj.com/site/about/guidelines.xhtml

possible dependence between the different withdrawal risks, cumulative incidence analyses will be presented (29).

The Haybittle-Peto approach will be employed for each interim analysis, with 99.9% confidence intervals calculated for interim analysis effect estimates. The final analysis will be undertaken at the end of the trial when all patients have a minimum two year follow-up data (six and a half years after the first patient is randomised) and 95% confidence intervals will be calculated.

QOL data will be analysed longitudinally to explore between treatments changes in scale scores over time, taking account of baseline QOL.

For the analysis of adverse reactions, all patients who received any amount of each study drug will be included in the safety analysis dataset in the treatment group they actually received. All adverse reactions and serious adverse reactions reported by the clinical investigators will be presented, identified by treatment group. Adverse reactions will be grouped according to a pre-specified coding system and tabulated. The number (and percentage) of patients experiencing each AR, and the number (and percentage) of occurrences of each AR will be presented. No formal statistical testing will be undertaken.

HEALTH ECONOMIC EVALUATION

For the health economic analyses, the perspective of the NHS and Personal Social Services will be adopted for costing purposes. It will be assessed whether levetiracetam or zonisamide as monotherapy in newly-treated focal epilepsy are cost-effective by estimating the incremental cost-utility and cost-effectiveness ratios relative to lamotrigine and to each other. The same approach will be used to compare levetiracetam and valproate for generalised-onset seizures. A cost consequence analysis (30) will be conducted to consider non-health benefits which are neither captured within the QALY calculation, nor in the cost-effectiveness analyses. Potential non-health benefits that will be measured include: social activity, time in work or school and patients' driving (captured in the NEWQOL battery). Additional non-health benefits (perceived stigma, control, and cognitive effects) will also be captured in the NEWQOL-6D (23).

Sensitivity analyses will be conducted to test the robustness of our findings. These analyses will be based on the observed distributions of outcome and costs to test whether, and to what extent, the incremental cost-utility and cost-effectiveness ratios are sensitive to key assumptions in the analysis. Uncertainty in parameter estimates will be addressed through the application of bootstrapping and the estimation of cost-effectiveness acceptability curves.

For peer review only - http://bmjopen.bmj.com/site/about/guidelines.xhtml

The estimated incremental cost per QALY will be compared with the threshold for cost-effectiveness operating in the UK, and the incremental cost per seizure avoided and per 12-month remission compared with results of other economic assessments of antiepileptic drugs (31, 32).

ETHICS and Dissemination

Ethics

The trial protocol complies with the SPIRIT reporting guidelines (33). All relevant trial documentation have been approved by the North West - Liverpool East Research Ethics Committee (Ref: 12/NW/0361). This trial falls within the remit of the EU Directive 2001/20/EC(34), transposed into UK law as the UK Statutory Instrument 2004 No 1031: Medicines for Human Use (Clinical Trials) Regulations 2004 as amended(35). SANAD-II has been registered with the MHRA and was granted a Clinical Trial Authorisation (CTA) for Notification prior to initiation.

Dissemination

The trial team plans to disseminate the SANADII results by presentations at national (e.g. ABN) and international (e.g. ILAE – European congress, or ILAE international Epilepsy Congress) meetings, as well as publications in leading peer reviewed journals and through patient and public involvement. Authorship of any documents will follow ICMJE Recommendations 2018.

DATA COLLECTION AND TRIAL MONITORING

Data collection

Data management for SANAD-II is performed by the CTRC at University of Liverpool. Participating centres will be expected to each maintain a file of essential trial documentation (Site File), which will be provided by the coordinating centre, and keep copies of all completed Case Report Forms (CRFs). Data collection will use paper CRFs and participant completed questionnaires.

Trial Monitoring

Trial Oversight Committees have been formed in relation to the monitoring. This includes an Independent Data and Safety Monitoring Committee (IDSMC), consisting of independent epilepsy experts and statisticians; A Trial Management Group (TMG) consisting of the Chief Investigator, trial manager, trial statisticians, sponsor representatives, and several principal investigators, and a Trial

For peer review only - http://bmjopen.bmj.com/site/about/guidelines.xhtml

Steering Committee (TSC) consisting of two independent epilepsy clinicians, an independent statistician and a lay representative, in addition to select members of the TMG.

Trial monitoring is informed by a risk assessment to determine the level and type of monitoring required for specific hazards. The IDSMC will monitor participant safety including any unexpected adverse reactions. Adverse reactions will be reported to the MHRA and to the Liverpool East Research Ethics Committee via annual Data and Safety Monitoring Reports (DSURs). The committees will also monitor recruitment rates and adherence to GCP.

Patient and Public Involvement (PPI) Statement

SANAD II was designed in collaboration with Epilepsy Action and has a PPI representative in the Trial Steering Committee.

TIME FRAME AND TRIAL STATUS

The study opened to recruitment in 2013 and completed recruitment in June 2017. Participants are followed-up for a minimum of 2 years and a maximum of 6.5 years. Follow-up visits and data collection will continue until June 2019.

Author Affiliations

University of Liverpool, University of Glasgow, Bangor University, University Hospital of Wales, Alder Hey Children's Health Park, Imperial College London

Acknowledgments

We would like to acknowledge the input of the wider SANAD-II team and to thank the research sites and participants for being involved in this study. We would like to specifically thank Epilepsy Action for their invaluable contribution throughout the trial.

Author Contributions

GS, PS, RA, JL, MJ, G Baker, MP, DH, PW, CTS and AM were involved in the trial design and the grant application preparation. SB, GS, G Burnside, CP, PS, RA, JL, MJ, G Baker, MP, DH, PW, CTS and AM were involved in the protocol development and protocol amendments throughout the trial. SB, CT and AM were responsible for the drafting of the manuscript. All authors were able to contribute to the draft and approved the final text.

For peer review only - http://bmjopen.bmj.com/site/about/guidelines.xhtml

Trial Sponsors

The SANADII trial is co-sponsored by the University of Liverpool (Sponsor Representative Alex Astor [sponsor@liverpool.ac.uk]) and The Walton Centre (Sponsor Representative Martin Wilby [RDI@thewaltoncentre.nhs.uk]).

Funding statement

This work is supported by the Health Technology Assessment Programme (HTA) of National Institute for Health Research (NIHR) grant number 09/144/09.

Competing Interests

None declared.

Provenance and peer review

The study, this protocol v8.0 dated 28/11/2018 and all superseded versions, and related trial documents have been approved by a registered Research Ethics Committee. The study was peer reviewed during the funding application process to the NIHR HTA programme.

Confidentiality and Data Sharing Statement

Identifiable details of those participating in the trial will remain confidential and accessible only to researchers at a patient's hospital site. The information collected by hospital site for the purpose of the study may be provided to researchers running other research studies. These researchers may be universities, NHS organisations or companies involved in health and care research in this country or abroad. The information will only be used by organisations and researchers to conduct research in accordance with the UK Policy Framework for Health and Social Care Research. This information will not identify any participants and will not be combined with other information in a way that could identify participants.

Upon completion of SANADII the trial results will be disseminated at scientific and/or medical conferences, through peer-reviewed publications, and will be made available on the study website.

Open Access

This is an Open Access article.

REFERENCES

For peer review only

For peer review only - http://bmjopen.bmj.com/site/about/guidelines.xhtml

1. Hauser WA HD. Epilepsy: frequency, causes and consequences. : New York: Demos Publications; 1990.
2. Holland P, Lane S, Whitehead M, Marson AG, Jacoby A. Labor market participation following onset of seizures and early epilepsy: Findings from a UK cohort. Epilepsia. 2009;50(5):1030-9.
3. Schachter SC. Quality of life for patients with epilepsy is determined by more than seizure control: the role of psychosocial factors. Expert Rev Neurother. 2006;6(1):111-8.
4. Marson AG, Al-Kharusi AM, Alwaidh M, Appleton R, Baker GA, Chadwick DW, et al. The SANAD study of effectiveness of carbamazepine, gabapentin, lamotrigine, oxcarbazepine, or topiramate for treatment of partial epilepsy: an unblinded randomised controlled trial. Lancet. 2007;369(9566):1000-15.
5. Marson AG, Al-Kharusi AM, Alwaidh M, Appleton R, Baker GA, Chadwick DW, et al. The SANAD study of effectiveness of valproate, lamotrigine, or topiramate for generalised and unclassifiable epilepsy: an unblinded randomised controlled trial. Lancet. 2007;369(9566):1016-26.
6. Commission on Classification and Terminology of the International League Against Epilepsy. Proposal for revised classification of epilepsies and epileptic syndromes. In: Epilepsy. CoCaTotILA, editor. Epilepsia1989 Jul-Aug. p. 389-99.
7. Bonnett L TSC, Williamson PR, Marson AG. Developing a Prognostic Model for Epilepsy Data. 31st Annual Conference of the International Society for Clinical Biostatisticians, ISCB; Montpellier2010. p. 145.
8. Brodie MJ, Perucca E, Ryvlin P, Ben-Menachem E, Meencke HJ, Levetiracetam Monotherapy Study G. Comparison of levetiracetam and controlled-release carbamazepine in newly diagnosed epilepsy. Neurology. 2007;68(6):402-8.
9. Trinka E, Marson AG, Van Paesschen W, Kalviainen R, Marovac J, Duncan B, et al. KOMET: an unblinded, randomised, two parallel-group, stratified trial comparing the effectiveness of levetiracetam with controlled-release carbamazepine and extended-release sodium valproate as monotherapy in patients with newly diagnosed epilepsy. J Neurol Neurosurg Psychiatry. 2013;84(10):1138-47.
10. Baulac M, Brodie MJ, Patten A, Segieth J, Giorgi L. Efficacy and tolerability of zonisamide versus controlled-release carbamazepine for newly diagnosed partial epilepsy: a phase 3, randomised, double-blind, non-inferiority trial. Lancet Neurol. 2012;11(7):579-88.
11. National Institute for Health and Clinical Excellence. The epilepsies: the diagnosis and management of the epilepsies in adults and children in primary and secondary care.; 2004.
12. Marson AG, Williamson PR, Hutton JL, Clough HE, Chadwick DW. Carbamazepine versus valproate monotherapy for epilepsy. Cochrane Database Syst Rev. 2000(3):CD001030.
13. Tudur Smith C, Marson AG, Williamson PR. Phenytoin versus valproate monotherapy for partial onset seizures and generalized onset tonic-clonic seizures. Cochrane Database Syst Rev. 2001(4):CD001769.
14. Glauser TA, Cnaan A, Shinnar S, Hirtz DG, Dlugos D, Masur D, et al. Ethosuximide, valproic acid, and lamotrigine in childhood absence epilepsy. N Engl J Med. 2010;362(9):790-9.
15. Morrow J, Russell A, Guthrie E, Parsons L, Robertson I, Waddell R, et al. Malformation risks of antiepileptic drugs in pregnancy: a prospective study from the UK Epilepsy and Pregnancy Register. J Neurol Neurosurg Psychiatry. 2006;77(2):193-8.
16. Meador KJ, Baker GA, Browning N, Clayton-Smith J, Combs-Cantrell DT, Cohen M, et al. Cognitive function at 3 years of age after fetal exposure to antiepileptic drugs. N Engl J Med. 2009;360(16):1597-605.
17. EMA. New measures to avoid valproate exposure in pregnancy endorsed: Member State representatives agree new restrictions and pregnancy prevention programme. 2018 [cited 2019 21st March]. Available from: https://www.ema.europa.eu/en/documents/press-release/new-measures-avoid-valproate-exposure-pregnancy-endorsed_en.pdf.

For peer review only - http://bmjopen.bmj.com/site/about/guidelines.xhtml

18. Jacoby A, Baker G, Smith D, Dewey M, Chadwick D. Measuring the impact of epilepsy: the development of a novel scale. Epilepsy Res. 1993;16(1):83-8.
19. Ravens-Sieberer U, Bullinger M. Assessing health-related quality of life in chronically ill children with the German KINDL: first psychometric and content analytical results. Qual Life Res. 1998;7(5):399-407.
20. Cramer JA, Westbrook LE, Devinsky O, Perrine K, Glassman MB, Camfield C. Development of the Quality of Life in Epilepsy Inventory for Adolescents: the QOLIE-AD-48. Epilepsia. 1999;40(8):1114-21.
21. Griebsch I, Coast J, Brown J. Quality-adjusted life-years lack quality in pediatric care: a critical review of published cost-utility studies in child health. Pediatrics. 2005;115(5):e600-14.
22. Prosser LA, Hammitt JK, Keren R. Measuring health preferences for use in cost-utility and cost-benefit analyses of interventions in children: theoretical and methodological considerations. Pharmacoeconomics. 2007;25(9):713-26.
23. Abetz L, Jacoby A, Baker GA, McNulty P. Patient-based assessments of quality of life in newly diagnosed epilepsy patients: validation of the NEWQOL. Epilepsia. 2000;41(9):1119-28.
24. Beecham J KM. Costing psychiatric interventions. In: G T, editor. Measuring Mental Health Needs. 2nd ed. London: Gaskell; 2001. p. 200-24.
25. NHS. User guide: reference costs 2017/18. 2018 [cited 2019 29th April]. Available from: https://improvement.nhs.uk/documents/1978/6_-_Reference_costs_2017-18_A_Guide_to_using_the_data.pdf.
26. BNF77. BMJ Group and Pharmaceutical Press; 2019.
27. Curtis LA. Unit Costs of Health and Social Care 2018. Personal Social Services Research Unit, University of Kent, Canterbury; 2018.
28. Chadwick D. Considerations on designing clinical trials to evaluate the place of new antiepileptic drugs in the treatment of newly diagnosed and chronic patients with epilepsy. 1998;39(7):799-803.
29. Williamson PR, Smith CT, Sander JW, Marson AG. Importance of competing risks in the analysis of anti-epileptic drug failure. Trials. 2007;8:12.
30. Mauskopf JA, Paul JE, Grant DM, Stergachis A. The role of cost-consequence analysis in healthcare decision-making. Pharmacoeconomics. 1998;13(3):277-88.
31. Connock M, Frew E, Evans BW, Bryan S, Cummins C, Fry-Smith A, et al. The clinical effectiveness and cost-effectiveness of newer drugs for children with epilepsy. A systematic review. Health Technol Assess. 2006;10(7):iii, ix-118.
32. Wilby J, Kainth A, Hawkins N, Epstein D, McIntosh H, McDaid C, et al. Clinical effectiveness, tolerability and cost-effectiveness of newer drugs for epilepsy in adults: a systematic review and economic evaluation. Health Technol Assess. 2005;9(15):1-157, iii-iv.
33. Chan AW, Tetzlaff JM, Altman DG, Laupacis A, Gotzsche PC, Krleza-Jeric K, et al. SPIRIT 2013 statement: defining standard protocol items for clinical trials. Ann Intern Med. 2013;158(3):200-7.
34. EU Directive 2001/20/EC.
35. The Medicines for Human Use (Clinical Trials) Regulations 2004 (SI 2004/1031).

For peer review only

For peer review only - http://bmjopen.bmj.com/site/about/guidelines.xhtml
